# Phase-Dependent Functionality and Defect-Induced Magnetism in Monolayer SnTe Polymorphs

**DOI:** 10.1021/acsami.6c07072

**Published:** 2026-06-03

**Authors:** Roya Kavkhani, Berna Akgenc Hanedar, Mehmet Cengiz Onbaşlı

**Affiliations:** † Graduate School of Sciences and Engineering (GSSE), 52979 Koç University, Rumelifeneri Yolu, Sarıyer, Istanbul 34450, Türkiye; ‡ Department of Physics, Kırklareli University, Kırklareli 39100, Türkiye; § Department of Electrical & Electronics Engineering, Koç University, Rumelifeneri Yolu, Sarıyer, Istanbul 34450, Türkiye; ∥ Department of Physics, Koç University, Rumelifeneri Yolu, Sarıyer, Istanbul 34450, Türkiye

**Keywords:** monolayer SnTe, phase engineering, defect engineering, two-dimensional materials, carrier mobility, magnetic semiconductors, infrared optoelectronics

## Abstract

Monolayer SnTe offers an unusual opportunity for functional materials design because its electronic and magnetic behavior can be tuned through two coupled control parameters: crystal phase and local defect chemistry. Here, first-principles calculations establish how these complementary design knobs govern the intrinsic and defect-driven properties of four SnTe polymorphs. Among the considered monolayers, cubic SnTe is identified as the ground-state phase, closely followed by γ-SnTe, while hexagonal and β′-SnTe are less favorable. This phase hierarchy is accompanied by strongly distinct electronic and transport regimes, with the HSE+SOC band gap spanning 0.35–1.84 eV, ultrahigh hole mobility emerging in cubic SnTe, strongly anisotropic electron transport appearing in hexagonal SnTe, and anisotropic hole transport found in β′-SnTe. Guided by this phase-stability landscape, defect engineering is then examined in cubic and γ-SnTe through substitutional doping and native vacancies. A clear phase-dependent defect response is revealed: dopants drive cubic SnTe toward effective metallicity, whereas γ-SnTe supports more selective outcomes, ranging from metallic to semiconducting behavior depending on dopant species. In particular, Mn is the most favorable magnetic dopant in both phases, and Mn-doped γ-SnTe combines strong spin polarization with a retained direct band gap, highlighting it as a promising magnetic semiconducting state. Together, these results show that phase selection defines the intrinsic band gap and transport landscape of monolayer SnTe, while defect engineering provides additional control over metallicity, magnetism, and carrier polarity. Monolayer SnTe therefore emerges as a tunable platform for infrared optoelectronic and spin-functional applications.

## Graphical abstract

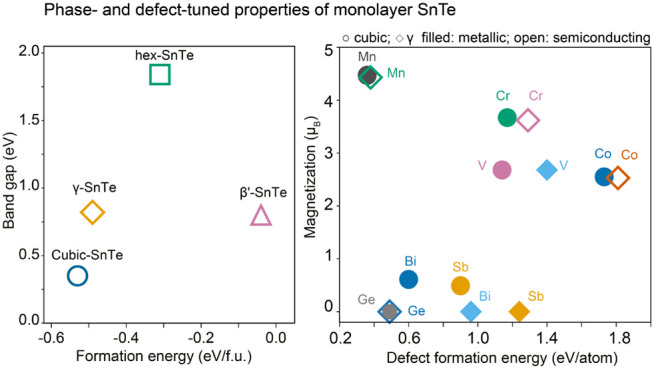

## 1 Introduction

Tin telluride (SnTe) is a narrow-gap IV–VI compound whose electronic and ferroic properties are highly sensitive to crystal structure, reduced dimensionality, and local chemical environment.^1–3^ In the two-dimensional (2D) limit, this sensitivity becomes even more pronounced because relatively small changes in bonding geometry and lattice symmetry can strongly modify structural stability, lattice dynamics, electronic structure, and carrier transport.^1,4–7^ This strong structure–property coupling makes monolayer SnTe especially attractive not only as a model system for low-dimensional condensed-matter physics but also as a candidate electronic and optoelectronic material in which functionality may be tailored through crystal phase and local chemistry. Its broader technological relevance is supported by the demonstrated promise of SnTe-based thin films and nanostructures in broadband photoresponse and related device functionalities.^8–15^ Realizing such potential in the monolayer limit, however, first requires identifying which structural forms are stable and how their atomic arrangements define the resulting physical properties.

This question is particularly important because thin-layer SnTe can adopt multiple competing structural phases.^14–16^ In such a system, polymorphism is not merely a structural detail; it can strongly reshape the energetic landscape and thereby govern the accessible electronic, vibrational, and transport properties. Establishing the relative stability of candidate monolayer phases is therefore a necessary starting point for phase-dependent functional design. Cohesive and formation energies assess thermodynamic favorability, phonon dispersions probe dynamical stability, and Raman-active modes together with simulated scanning tunneling microscopy (STM) images provide experimentally relevant structural fingerprints. Combined with electronic band structures and intrinsic carrier transport, these quantities enable a direct link between atomic arrangement and measurable functionality. Capturing this link, however, requires a unified comparison across the relevant polymorphs rather than isolated treatment of individual phases.

Despite this need, previous first-principles studies of monolayer SnTe have mostly focused on selected phases or on individual properties, leaving a broader phase-resolved picture incomplete.^17–20^ Yang et al. compared the thermoelectric behavior of five monolayer SnTe allotropes and assessed their stability through phonon spectra, MD simulations, and binding energies,^17^ but a more comprehensive framework remains lacking, particularly one that unifies energetic stability, lattice dynamics, electronic structure, intrinsic carrier transport, and experimentally accessible structural fingerprints within a single study. This missing framework is important because phase selection in monolayer SnTe is expected to do more than determine crystal geometry alone; it may also redefine the intrinsic band gap, switch the dominant transport regime, and alter how the monolayer responds to local chemical perturbations. Among these consequences, charge transport is especially significant because it provides one of the clearest links between phase selection and potential electronic functionality.

Charge transport is a central consideration in SnTe because carrier mobility strongly influences the suitability of a material for electronic and optoelectronic applications. Experimental studies on bulk and thin-film SnTe have reported a wide range of mobilities, reflecting strong sensitivity to crystallinity, carrier density, temperature, defect concentration, and structural quality.^21–24^ In the cubic phase, high-quality epitaxial SnTe films can exhibit especially large mobilities, including approximately 2000 cm^2^ V^–1^ s^–1^ at 15 K for SnTe grown on Bi_2_Te_3_ buffer layers,^25^ and about 992 cm^2^ V^–1^ s^–1^ at 4 K in SnTe/CdTe heterostructures.^23^ By contrast, polycrystalline or room-temperature samples typically show much lower values, often below 100 cm^2^ V^–1^ s^–1^,^26^ highlighting the strong influence of extrinsic scattering and disorder. For this reason, theoretical estimates of intrinsic mobility are particularly useful because they isolate the effect of the underlying crystal phase through band-edge effective mass, elastic response, and carrier–phonon coupling. A phase-resolved transport analysis can therefore reveal whether different monolayer SnTe polymorphs correspond to fundamentally distinct intrinsic conduction regimes. Once the intrinsic phase dependence is clarified, the next issue is whether it can be further modulated through local chemical modification.

Beyond phase selection, the properties of SnTe can be further tuned through point defects and chemical substitution. Native vacancies, particularly Sn vacancies, play a major role in determining carrier polarity and concentration in SnTe-based systems, while extrinsic dopants can strongly modify the near-Fermi-level electronic structure and may also induce magnetic, ferroic, thermoelectric, or transport-related responses depending on the dopant species.^2,27–31^ More broadly, point-defect engineering has emerged as an important route for tuning the behavior of 2D semiconductors, since vacancies and substitutional impurities can substantially alter the electronic structure, carrier type, and magnetic behavior depending on the host lattice and defect chemistry.^32,33^ For example, studies on defected single-layer GeSe have shown that vacancies and substitutional defects can strongly modify the electronic and magnetic properties of atomically thin materials,^32^ while related first-principles work on BeN_4_-based monolayers has demonstrated that point defects can induce pronounced defect-dependent changes in electronic response and functionality.^33^ In addition, recent reviews have emphasized that reliable interpretation of defect energetics and defect-induced electronic behavior requires careful first-principles modeling and reproducible defect-simulation protocols.^34,35^ These considerations are especially relevant in the 2D limit, where reduced coordination and broken local symmetry can enhance the impact of local perturbations. However, previous DFT studies of defects and dopants in SnTe have largely focused on bulk rocksalt SnTe or on individual monolayer structures rather than examining how defect response itself varies across different monolayer phases.^2,29,30,36–38^ As a result, it remains unclear whether defect engineering affects all SnTe polymorphs in a similar manner or whether its role is itself phase dependent. This distinction is crucial because phase selection and defect engineering are likely to provide complementary control knobs over functionality: the former defines the intrinsic electronic and transport landscape, whereas the latter offers additional control over carrier type, metallicity, and magnetic response. A unified treatment of these two factors is therefore needed to clarify how monolayer SnTe can be functionally tailored within a single material platform.

In this work, we present a comprehensive first-principles study of four monolayer SnTe polymorphs, namely the cubic, γ, hexagonal, and β′ phases. We first establish their relative stability and intrinsic phase-dependent functionality through optimized structures, cohesive and formation energies, electronic band structures, simulated STM images, phonon dispersions, Raman spectra, and intrinsic carrier mobilities. Guided by this phase comparison, we then focus on the two most favorable phases, cubic and γ-SnTe, to investigate how substitutional dopants and intrinsic vacancies modify their electronic and magnetic properties. Specifically, substitution of Sn by Bi, Sb, Mn, Ge, Cr, V, and Co, as well as Sn and Te vacancies, have been examined separately, and defect formation energies are evaluated to determine the relative favorability of the resulting configurations. By combining polymorph stability, experimentally relevant structural fingerprints, intrinsic transport analysis, and defect-driven electronic and magnetic tuning within a single framework, this work shows that crystal phase and local chemical modification act as complementary routes for controlling the behavior of monolayer SnTe. It therefore establishes phase selection and defect engineering as a coherent design strategy for tailoring its functional properties Figure 1.

**Figure 1:**
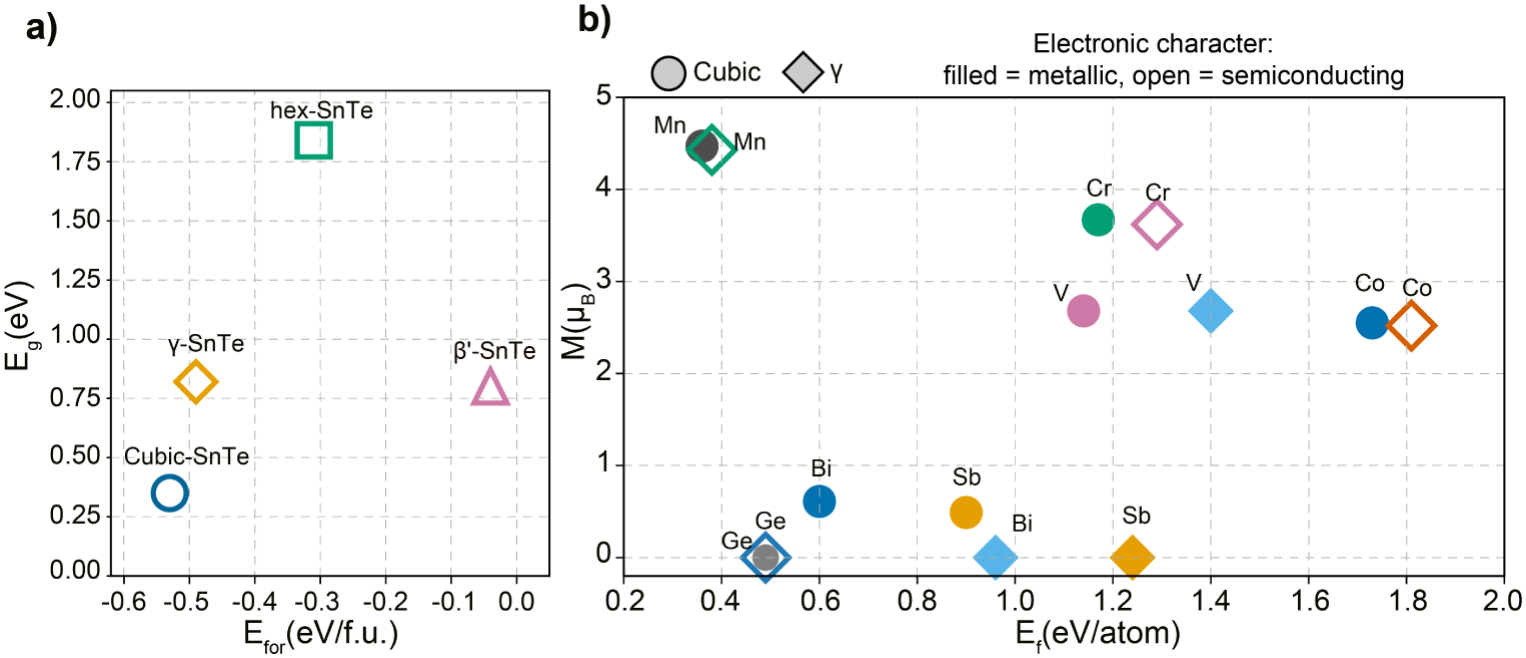
Structure–property maps summarizing the phase-dependent pristine electronic response of monolayer SnTe and the defect-induced magnetic behavior in cubic- and γ-SnTe. (a) Correlation between formation energy, *E*_for_, and band gap (calculated using HSE+SOC), *E_g_*, for the pristine polymorphs. (b) Magnetic moment, *M*, as a function of defect formation energy, *E_f_*, for substitutionally doped cubic- and γ-SnTe. Marker shape denotes the host phase, with circles representing cubic SnTe and diamonds representing γ-SnTe. Filled symbols indicate metallic behavior, whereas open symbols indicate semiconducting behavior.

## 2 Computational Methodology

First-principles calculations were carried out within the framework of density functional theory (DFT) using the projector augmented-wave (PAW) method, as implemented in the Vienna *Ab initio* Simulation Package (VASP).^39,40^ The plane-wave kinetic-energy cutoff was set to 500 eV.^41^ Exchange and correlation were described within the generalized gradient approximation using the Perdew–Burke–Ernzerhof (PBE) functional,^42^ and van der Waals interactions were included through Grimme’s DFT-D2 correction.^43^ The valence configurations were Sn-5*s* ^2^5*p* ^2^, Te-5*s* ^2^5*p* ^4^, Bi-6*s* ^2^6*p* ^3^, Sb-5*s* ^2^5*p* ^3^, Mn-3*d* ^6^4*s* ^1^, Ge-4*s* ^2^4*p* ^2^, Cr-3*d* ^5^4*s* ^1^, V-3*p* ^6^4*s* ^1^3*d* ^4^, and Co-3*d* ^8^4*s* ^1^. Electronic self-consistency and ionic relaxation were converged to 10^–5^ eV and 0.01 eV Å^–1^, respectively. A vacuum spacing of at least 15 Å was introduced along the out-of-plane direction to avoid spurious interactions between periodically repeated images. Four monolayer SnTe polymorphs, cubic, γ, hexagonal, and β′, were considered. Brillouin-zone sampling for the primitive cells employed a Γ-centered 12 × 12 × 1 *k*-point mesh.

Based on the calculated relative stabilities, cubic and γ-SnTe, corresponding to the two lowest-energy phases, were selected for defect calculations. Point defects were introduced as substitutional dopants and native vacancies. Specifically, Bi, Sb, Mn, Ge, Cr, V, and Co were substituted on the Sn site, while intrinsic defects were modeled by removing either one Sn atom or one Te atom. Defective cubic SnTe was modeled using a 2 × 2 × 1 supercell containing 24 Sn and 24 Te atoms, whereas defective γ-SnTe was described using a 3 × 3 × 1 supercell containing 18 Sn and 18 Te atoms. In each case, a single dopant atom or vacancy was introduced to represent an isolated point defect. These supercells provide minimum separations of approximately 12.58 Å for cubic SnTe and 13.49 Å for γ-SnTe between periodic defect images, thereby reducing artificial defect–defect interactions.

To account for crystallographic symmetry, all inequivalent defect sites were examined. In cubic SnTe, four symmetry-distinct groups were identified for both Sn and Te sublattices, whereas in γ-SnTe two inequivalent groups were considered. These configurations are illustrated in Figures 2 and 3. For each dopant and vacancy type, all inequivalent structures were fully optimized to determine the lowest-energy configuration. Both nonspin-polarized and spin-polarized calculations were performed for every case to identify the preferred electronic and magnetic ground state. For defect supercells, Brillouin-zone integration was carried out using a Γ-centered 4 × 4 × 1 *k*-point mesh.

**Figure 2:**
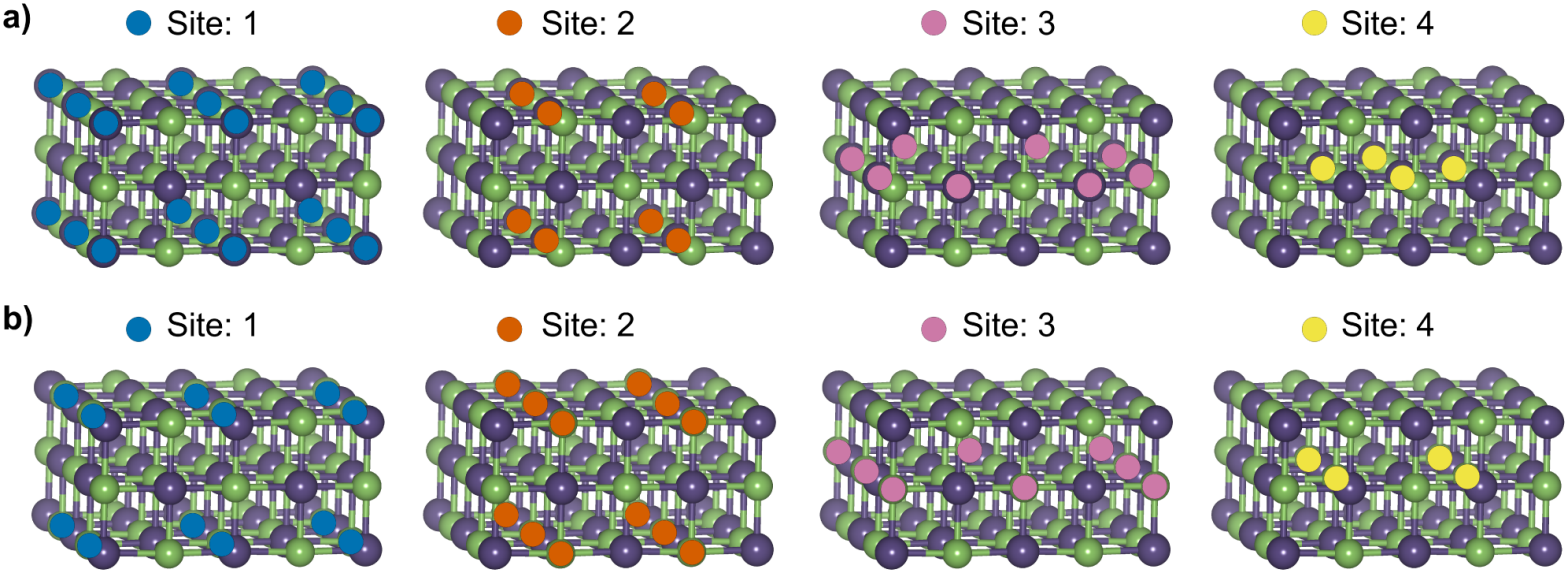
Symmetry-equivalent Sn and Te sites in the cubic SnTe 2 × 2 × 1 supercell. Purple and green spheres represent Sn and Te atoms, respectively, while blue, orange, pink, and yellow spheres indicate the four site groups considered for defect or dopant placement. (a) Sn sites and (b) Te sites.

**Figure 3:**
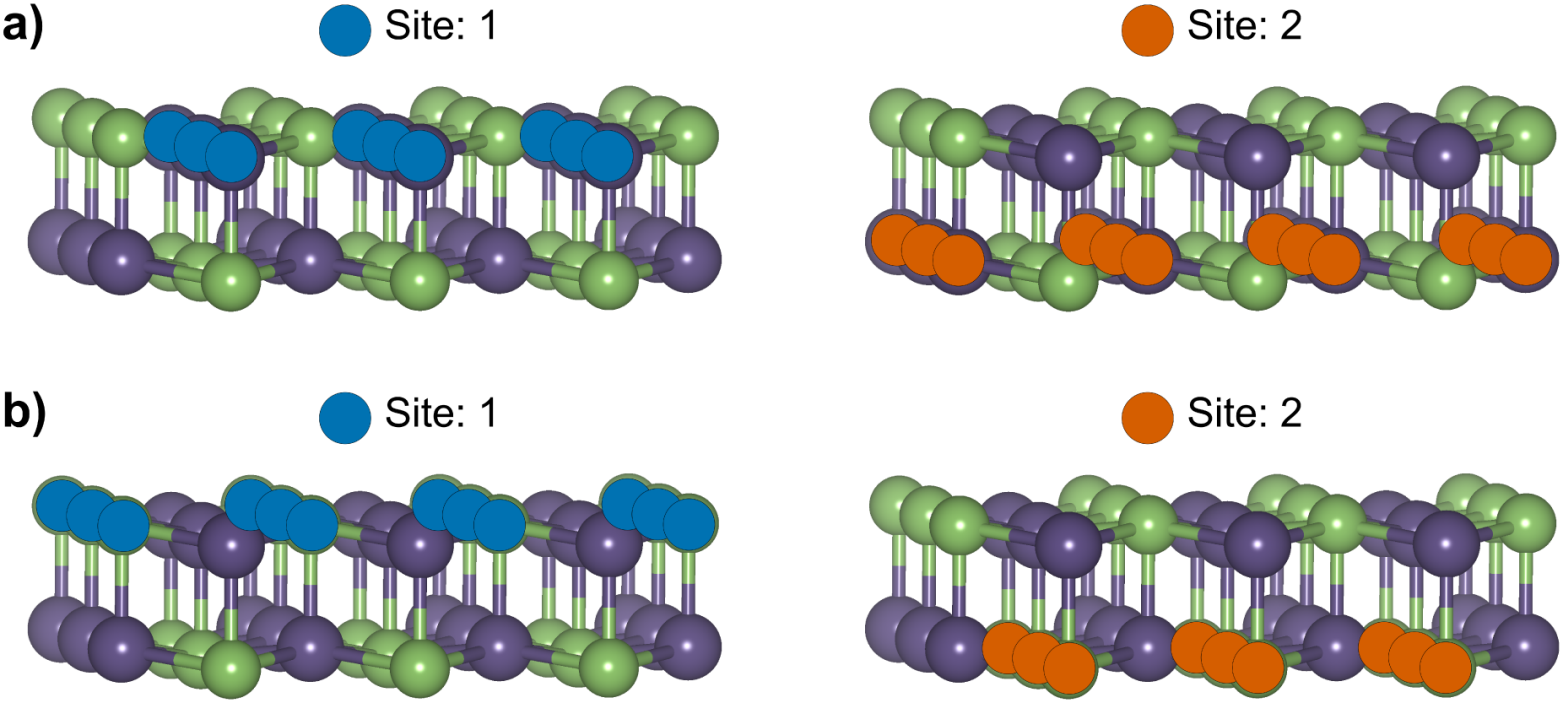
Equivalent Sn and Te atomic sites in the γ-SnTe 3 × 3 × 1 supercell. Purple and green spheres represent Sn and Te atoms, respectively, while blue and orange spheres highlight the two symmetry-equivalent site groups considered for defect or dopant incorporation. (a) Sn sites and (b) Te sites.

Electronic band structures were subsequently calculated for both pristine and defective systems. For the pristine monolayers, band structures were obtained at both the PBE+SOC and HSE+SOC levels in order to assess the role of exchange–correlation treatment while explicitly including spin–orbit coupling. For the defect-containing supercells, band structures were computed at the PBE+SOC level. Because hybrid-functional calculations for large defect supercells are substantially more computationally demanding, the defect-containing systems were treated within a single and internally consistent PBE+SOC framework. Accordingly, the absolute defect-state energetics may depend on the exchange-correlation functional, although the qualitative phase- and defect-dependent trends discussed here remain informative. Owing to Brillouin-zone folding in the supercell representation, band unfolding was performed for all defective cubic and γ-SnTe systems to recover the effective band dispersion in the primitive-cell Brillouin zone and to enable direct comparison with the pristine phases. The primitive-cell, folded supercell, and unfolded band structures are presented in Figure S1 in the Supporting Information (SI).

The thermal stability of the pristine and defect-containing monolayers was further evaluated by *ab initio* molecular dynamics (AIMD) simulations at 300 K. For the pristine phases, 4 × 4 × 1 supercells were used with a time step of 1 fs and a total simulation time of 5 ps, as shown in Figure S2. For defective cubic and γ-SnTe, the optimized defect supercells were further expanded to 2 × 2 × 1 and studied under identical conditions. The corresponding results are shown in Figures S3 and S4.

Phonon dispersions of the pristine monolayer phases were calculated using the small-displacement method as implemented in the PHON code. For force-constant calculations, the electronic energy convergence threshold was tightened to 10^–8^ eV. Each atom in the primitive cell was displaced by 0.01 Å, and the resulting force constants were used to construct the dynamical matrix, from which phonon frequencies and vibrational modes were obtained by direct diagonalization. The vibrational properties were further analyzed through off-resonant Raman activities of the Γ-point phonon modes. In this procedure, the phonon eigenvectors were first obtained from finite-displacement calculations in VASP, after which the derivatives of the macroscopic dielectric tensor with respect to the normal-mode coordinates were evaluated to determine the Raman activities.

Simulated scanning tunneling microscopy (STM) images were generated for the pristine monolayer phases within the Tersoff–Hamann approximation.^44^ Within this framework, the tunneling current is proportional to the energy-integrated local density of states evaluated at a constant tip–sample distance of approximately 2 Å above the surface. The integration window was defined by the applied bias voltage: positive sample bias probes unoccupied states above the conduction-band minimum, whereas negative sample bias probes occupied states below the valence-band maximum. This approach was applied to cubic, γ, hexagonal, and β′ monolayer SnTe.

## 3 Results and Discussion

### 3.1 Phase-Dependent Structural, Energetic, Electronic, and STM Properties of SnTe

Figure 1 summarizes the phase- and defect-dependent property landscape of monolayer SnTe and provides a useful framework for the discussion that follows. In the pristine limit, shown in Figure 1a, the four polymorphs are clearly separated in the formation-energy–band gap plane, demonstrating that crystal phase selection alone generates substantial variation in both thermodynamic stability and intrinsic electronic structure. The cubic phase combines the lowest formation energy with the smallest band gap, whereas γ-SnTe remains energetically competitive while exhibiting a distinctly larger gap, and the hexagonal and β′ phases shift toward wider-gap but less favorable regions. Although the absolute magnitudes of the calculated gaps may retain some dependence on the underlying exchange–correlation treatment even at the hybrid-functional level, the relative gap hierarchy is well resolved, indicating that the phase-dependent contrast in electronic response is robust. In the doped systems, shown in Figure 1b, substitutional defects introduce an additional degree of control over both magnetic and electronic behavior. Specifically, the magnetic moment can be tuned from nearly zero to values exceeding 4 μ_B_, while the corresponding electronic character may remain semiconducting or evolve toward metallicity depending on the dopant species and host phase. It should also be noted that the energy axes in Figure 1a and b correspond to different thermodynamic quantities, namely the formation energies of the pristine phases and the defect formation energies of the substituted systems, respectively; consequently, their numerical values, including their signs, are not intended for direct comparison. Taken together, these two panels capture the central materials-design picture established in this work: crystal polymorphism governs the intrinsic stability, band gap hierarchy, and transport regime of monolayer SnTe, whereas defect engineering broadens the accessible functionality toward tunable metallic, semiconducting, and spin-polarized states. This combined phase–defect landscape provides a realistic basis for infrared optoelectronic, anisotropic transport, and spin-functional device concepts within a single SnTe material family.

The cubic, γ, hexagonal, and β′ SnTe monolayer polymorphs are benchmarked within a consistent first-principles framework by comparing their optimized structures, energetic stability, electronic band structures, and simulated scanning tunneling microscopy (STM) contrast. The energetic preference among phases is quantified using the formation energy, $E_{\mathrm{for}}^{\left(\mathrm{f.u.}\right)}$ , and cohesive energy, $E_{\mathrm{coh}}^{\left(\mathrm{f.u.}\right)}$ , reported per formula unit (f.u.). $E_{\mathrm{for}}^{\left(\mathrm{f.u.}\right)}$ measures the thermodynamic driving force for forming SnTe from elemental reservoirs, whereas $E_{\mathrm{coh}}^{\left(\mathrm{f.u.}\right)}$ captures the intrinsic bonding strength within the freestanding monolayer:

1 $$E_{\mathrm{for}}^{\left(\mathrm{f.u.}\right)}=E_{\mathrm{SnTe}}-n_{\mathrm{Sn}}\mu_{\mathrm{Sn}}-n_{\mathrm{Te}}\mu_{\mathrm{Te}}$$

2 $$E_{\mathrm{coh}}^{\left(\mathrm{f.u.}\right)}=n_{\mathrm{Sn}}E_{\mathrm{Sn}}+n_{\mathrm{Te}}E_{\mathrm{Te}}-E_{\mathrm{SnTe}}$$

In eqs 1–2, *E*_SnTe_ denotes the total energy of the SnTe unit cell for a given monolayer phase, and *n*_Sn_ (*n*_Te_) is the number of Sn (Te) atoms in that cell. *E*_Sn_ and *E*_Te_ are the total energies of isolated Sn and Te atoms, respectively. The elemental chemical potentials μ_Sn_ and μ_Te_ are referenced to the corresponding bulk phases and are computed as μ_atom_ = *E*_bulk_/*N*, where *E*_bulk_ is the total energy of the bulk reference cell containing *N* atoms. The relaxed geometries of the cubic, γ, hexagonal, and β′ SnTe monolayers are presented in Figure 4a,d,g,j, and the extracted structural descriptors together with the stability metrics are compiled in Table 1. All pristine SnTe polymorphs investigated here were found to be nonmagnetic, with zero total magnetic moment.

**Figure 4:**
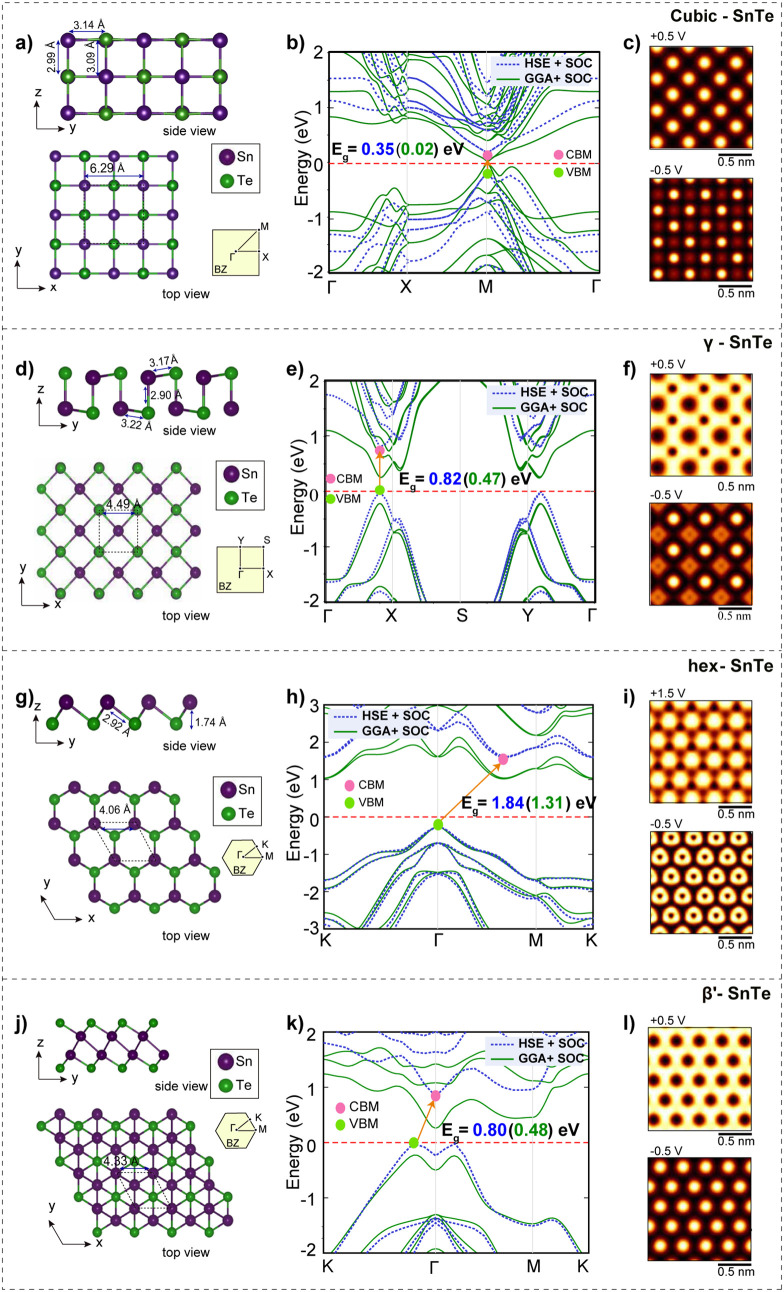
(a–c) Cubic SnTe, (d–f) γ-SnTe, (g–i) hexagonal SnTe, and (j–l) β′-SnTe. For each phase, the optimized crystal structures (side and top views), simulated STM images at the indicated bias voltages, and electronic band structures calculated using GGA+SOC and HSE+SOC along high-symmetry directions are shown, with the corresponding band gaps *E_g_* marked.

**Table 1:** Summary of the Calculated Energetic, Structural, Electronic, and Vibrational Properties of Pristine Monolayer SnTe Polymorphs, Including *E*_coh_, *E*_for_, Lattice Constants (*a*, *b*), Sn–Te Bond Length (*d*_Sn–Te_), Layer Thickness (*d*_thickness_), Band Gap (*E_g_*), and Raman Mode Frequencies^a^

Phase	*E*_coh_ (eV/f.u.)	*E*_for_ (eV/f.u.)	*a* (Å)	*b* (Å)	*d*_Sn–Te_ (Å)	*d*_thickness_ (Å)	*E*_*g*_ (eV)	Raman modes (cm^–1^)
Cubic	6.63	–0.53	6.29	6.29	3.07	6.20	0.35 (Dir)	48.5, 79.85, 92.9, 142.5
γ	6.59	–0.49	4.49	4.49	3.22	2.90	0.82 (Dir)	33.7, 59.4, 137.1
Hexagonal	6.41	–0.31	4.06	4.06	2.92	1.74	1.84 (Indir), 2.56 (Dir)	130.2, 190.5
β′	6.15	–0.04	4.33	4.33	2.96	5.42	0.80 (Indir), 1.13 (Dir)	42.1, 46.2, 118.1, 168.6

a Direct and indirect band gaps are indicated by Dir and Indir, respectively.

Among these structures, the cubic phase is energetically the most favorable. As shown in Figure 4a, the cubic SnTe monolayer relaxes into a highly symmetric, rocksalt-derived square lattice in the plane, with an optimized in-plane lattice constant of *a* = 6.29 Å. While the top view preserves this ideal square symmetry, the side view reveals the effect of reduced dimensionality on the bonding geometry: the in-plane nearest-neighbor Sn–Te distance is 3.14 Å, whereas the out-of-plane nearest-neighbor separations are slightly shorter (2.99–3.09 Å), yielding an overall monolayer thickness of 6.20 Å between the outermost atomic planes.

This compact and symmetric bonding configuration underpins the energetic stability of the cubic phase. Specifically, it exhibits both the largest cohesive energy (6.63 eV/f.u.) and the most negative formation energy (−0.53 eV/f.u.) among the considered polymorphs, signifying strong internal bonding and a pronounced thermodynamic driving force for formation from elemental Sn and Te. Consequently, the cubic monolayer emerges as the thermodynamic ground state within this set of SnTe polymorphs and is therefore expected to be the most readily accessible phase under equilibrium or near-equilibrium growth conditions.

The γ phase, while structurally related to the cubic lattice through its square-derived topology, introduces buckling and modified Sn–Te bond geometries. As shown in Figure 4d, γ-SnTe adopts a buckled square-like lattice composed of alternating Sn and Te atoms forming a two-dimensional network, with symmetric in-plane lattice constants (*a* = *b* = 4.49 Å) and no Sn–Sn bonding. The in-plane Sn–Te bond length is 3.22 Å, and the vertical corrugation produces a reduced monolayer thickness of 2.90 Å.

Despite this reduced symmetry, the energetic stability of the γ phase remains close to that of the cubic structure, with a cohesive energy of 6.59 eV/f.u. and a formation energy of −0.49 eV/f.u. (Table 1). This small energetic separation indicates strong competition between the cubic and γ motifs. Consequently, γ-SnTe may coexist with, or locally replace, the cubic phase in ultrathin films where strain, substrate registry, or kinetic effects during growth play a significant role.^16^

In contrast, as shown in Figure 4g, the hexagonal phase adopts a honeycomb-like lattice with reduced coordination and lower packing density, in which Sn and Te atoms occupy alternating sublattices. The in-plane lattice constants are smaller (*a* = *b* = 4.06 Å) than in the square-derived phases. The shorter Sn–Te bond length (2.92 Å) and reduced monolayer thickness (1.74 Å) reflect a more weakly connected network with diminished vertical corrugation and no Sn–Sn bonding. Consistently, the hexagonal polymorph shows reduced thermodynamic stability relative to the cubic and γ phases (Table 1), suggesting that it is more likely to appear as a metastable phase stabilized by growth kinetics or substrate effects.^45^

The β′ phase exhibits the lowest symmetry and the weakest thermodynamic driving force for formation. As illustrated in Figure 4j, β′-SnTe relaxes into a distorted lattice composed of alternating buckled Sn and Te sublayers forming a corrugated network. The optimized in-plane lattice constant is 4.33 Å, with a Sn–Te bond length of 2.96 Å, while the monolayer thickness reaches 5.42 Å. Unlike the γ and hexagonal structures, the β′ phase exhibits direct Sn–Sn bonding, reflecting a distinct local bonding environment. Although its cohesive energy remains sizable (6.15 eV/f.u.), the formation energy is only weakly negative (−0.04 eV/f.u.), indicating a marginal thermodynamic preference for formation from elemental Sn and Te. This near-zero formation energy suggests that β′-SnTe is unlikely to form under equilibrium conditions but may be stabilized under nonequilibrium growth or via strain/defect effects.^24^ Although the hexagonal and β′ phases are less favorable than cubic- and γ-SnTe under the present freestanding conditions, such higher-energy polymorphs may still become experimentally accessible through substrate interactions, epitaxial strain, kinetic growth pathways, or defect-assisted stabilization in ultrathin films.

Figure 4b,e,h, and k present the electronic band structures of the cubic, γ, hexagonal, and β′ SnTe polymorphs along the high-symmetry paths of their corresponding 2D Brillouin zones. These results reveal the distinct phase-dependent electronic features of monolayer SnTe, including differences in band gap magnitude, dispersion near the band edges, and the reciprocal-space location of the valence- and conduction-band extrema. Such variations highlight the strong influence of crystal structure on the intrinsic electronic character of each phase.

Figure 4c,f,i, and l present the simulated STM topographies of the cubic, γ, hexagonal, and β′ SnTe polymorphs, providing surface-sensitive real-space fingerprints of each phase. Within the Tersoff–Hamann approximation, the tunneling current at a given sample bias is proportional to the LDOS integrated over the corresponding energy window, with negative bias predominantly probing occupied states below the Fermi level and positive bias probing unoccupied states above it.^44^ As a result, the STM contrast and its bias dependence are controlled by the near-edge electronic structure, including the band gap magnitude, the reciprocal-space location of the band extrema, and the orbital or sublattice character of the band-edge states. The simulated images therefore offer a direct real-space manifestation of the underlying electronic structure. When analyzed together with the calculated band structures, these STM patterns establish a clear correspondence between the electronic fine structure of each phase and its experimentally observable surface characteristics, thereby providing a practical basis for phase identification in ultrathin SnTe films through future STM/STS measurements.^1,26^

For the cubic phase, the electronic band structure is plotted along the Γ–X–M−Γ path of the square Brillouin zone (Figure 4b). The bands exhibit an almost closed direct gap at the *M* point within GGA+SOC, with *E*_*g*_ ≈ 0.02 eV, which opens to *E*_*g*_ ≈ 0.35 eV when hybrid exchange is included. Although an explicit band inversion is not clearly resolved in the present calculation, the cubic phase remains of topological interest because bulk SnTe is a prototypical topological crystalline insulator.^46,47^ In SnTe-derived ultrathin films, however, the topological signature can depend strongly on thickness, since hybridization between opposite surfaces may open a gap and obscure Dirac-like features.^47^ Therefore, the absence of unambiguous topological signatures at the present thickness does not necessarily exclude topological crystalline insulator–related behavior, but may instead reflect finite-thickness effects. This near-gapless electronic structure implies a finite LDOS in close proximity to the Fermi level on both sides of the gap. As a consequence, the simulated STM images (Figure 4c) display a relatively symmetric and weakly bias-dependent contrast, consistent with semimetallic or narrow-gap semiconducting behavior. Such characteristics indicate that transport and spectroscopic measurements should detect finite spectral weight near the Fermi level, particularly at low temperatures.

The γ phase band structure is shown along the Γ–Y–S–X−Γ path of its rectangular Brillouin zone (Figure 4e). A well-defined direct band gap appears along the X−Γ direction, with *E*_*g*_ = 0.47 eV at the GGA+SOC level, increasing to 0.82 eV with HSE+SOC. This clear energetic separation between valence- and conduction-band states at a specific zone-edge point leads to a strong asymmetry in the LDOS probed under opposite bias polarities. Accordingly, the STM topographies of the γ phase (Figure 4f) exhibit pronounced bias-dependent contrast, with different atomic sublattices selectively enhanced under positive and negative bias. This behavior directly reflects the distinct orbital character of the band-edge states near *X* and provides a robust surface fingerprint of the underlying electronic structure.

For the hexagonal phase, the electronic bands are plotted along the *K*–Γ–M–K path of the hexagonal Brillouin zone (Figure 4h). This phase exhibits the largest band gap among the four polymorphs and an indirect-gap character, with the valence band maximum located at Γ and the conduction band minimum shifted along the Γ–M direction. At the HSE+SOC level, the indirect band gap is *E*_*g*_ = 1.84 eV, while the direct gap is larger (*E*_*g*_ = 2.56 eV). The wide indirect gap strongly suppresses the LDOS near the Fermi level, leading to a reduced tunneling probability within comparable bias windows. Consequently, the corresponding STM images (Figure 4i) show weaker and more spatially uniform contrast, consistent with insulating or wide-gap semiconducting behavior.

The β′ phase band structure is shown along the *K*–Γ–M–K path of its distorted Brillouin zone (Figure 4k). This phase occupies an intermediate electronic regime, exhibiting an indirect band gap of moderate magnitude, with the CBM at Γ and the VBM slightly displaced along the Γ–K direction. The band gap is *E*_*g*_ = 0.48 eV at the GGA+SOC level and increases to 0.80 eV when hybrid exchange is included. This intermediate electronic structure gives rise to STM contrast patterns (Figure 4l) that are more complex than those of the cubic phase but less strongly bias-asymmetric than in the γ phase. The observed contrast reflects the combined effects of reduced lattice symmetry, structural distortion, and mixed orbital contributions near the band edges.

Overall, the phase-dependent STM contrast across all four SnTe polymorphs can be consistently interpreted as a direct consequence of their electronic band structures. Differences in band gap magnitude, band-edge position in reciprocal space, and orbital character determine the LDOS distribution near the Fermi level, which in turn governs the bias-dependent STM response. This combined analysis establishes a physically grounded and experimentally actionable framework for identifying SnTe monolayer phases through joint consideration of band dispersion, tunneling spectroscopy, and surface-sensitive imaging.

### 3.2 Phase-Dependent Vibrational Properties of SnTe: Phonon and Raman Analysis

Figure 5 compares the lattice dynamics of four monolayer SnTe polymorphs by pairing phonon dispersion relations (stability and vibrational spectrum across the Brillouin zone) with the corresponding off-resonant Raman spectra at the Γ point (experimentally accessible fingerprints of zone-center modes).^48,49^ In all four cases (cubic, γ, hexagonal, and β′), the absence of imaginary phonon frequencies indicates dynamical stability of the relaxed structures at 0 K within the harmonic approximation.^48^ Near Γ, the acoustic branches show the expected linear in-plane modes and a quadratic flexural (ZA) branch characteristic of free-standing 2D sheets.^50^

**Figure 5:**
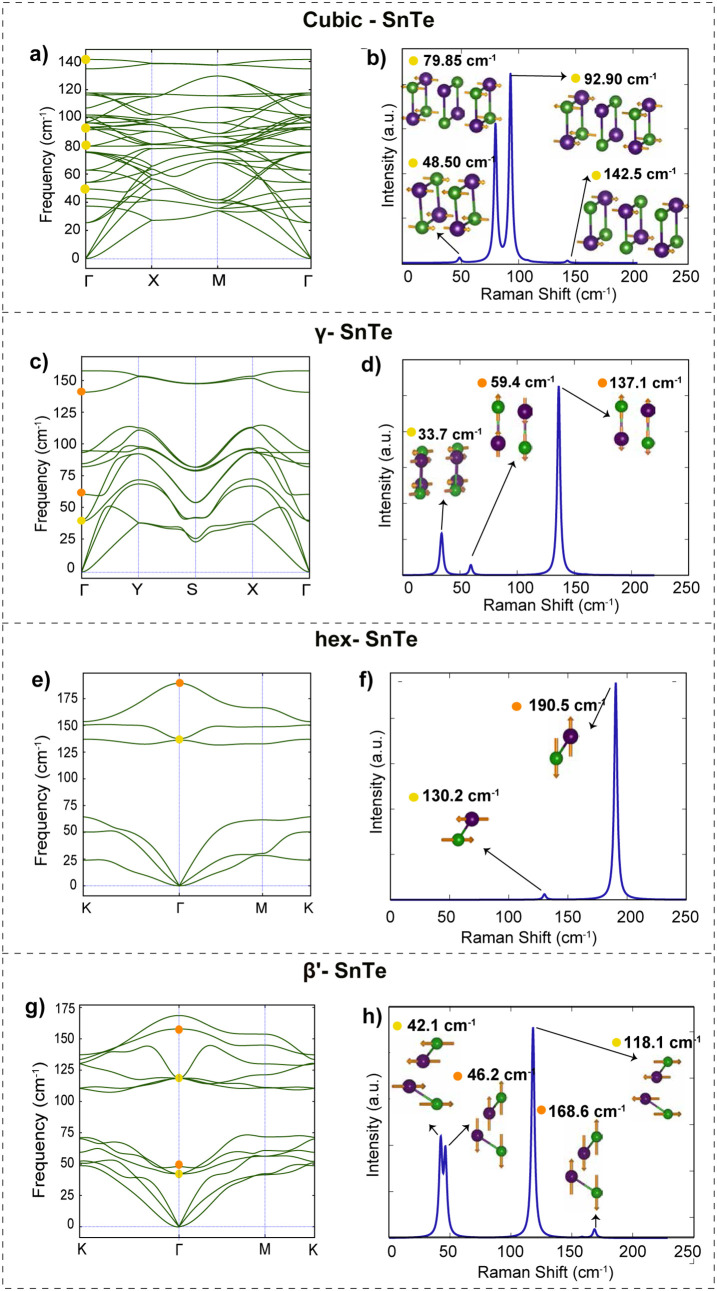
(a, b) Phonon dispersion relations and corresponding Raman spectra of cubic SnTe, (c, d) γ-SnTe, (e, f) hexagonal SnTe, and (g, h) β′-SnTe. The phonon spectra are plotted along the high-symmetry directions of the Brillouin zone, while the Raman spectra highlight the dominant vibrational modes with their characteristic frequencies indicated.

For the cubic phase (Figure 5a–b), the phonon dispersion exhibits well-separated acoustic and optical branches with no imaginary frequencies throughout the Brillouin zone, confirming the dynamical stability of cubic SnTe. The optical phonon modes extend up to approximately 140 cm^–1^, lower than those of the hexagonal and β′ phases, reflecting the high symmetry and relatively isotropic bonding environment of the rocksalt lattice. The corresponding Raman spectrum contains four main peaks at 48.5, 79.85, 92.90, and 142.5 cm^–1^. All Raman-active modes are dominated by in-plane atomic displacements, consistent with the symmetry-imposed selection rules at the Γ point.^49^ The lowest-frequency mode at 48.5 cm^–1^ originates from collective in-plane lattice vibrations with comparatively weak relative Sn–Te motion, resulting in low Raman intensity. The modes at 79.85 cm^–1^ and 92.90 cm^–1^ correspond to in-plane optical vibrations in which Sn and Te atoms move oppositely within the plane, producing strong modulation of the Sn–Te bond polarizability; among them, the 92.90 cm^–1^ mode exhibits the largest relative in-plane displacement and thus dominates the Raman response. The higher-frequency mode at 142.5 cm^–1^ also involves in-plane vibrations, but with more localized atomic motion, leading to a weaker Raman signature.

For the γ phase (Figure 5c–d), the optical phonon modes extend up to ∼150 cm^–1^, lower than those of the β and hexagonal phases, indicating comparatively weaker restoring forces. Three Raman-active modes are observed at 33.7 cm^–1^, 59.4 cm^–1^, and 137.1 cm^–1^. The lowest-frequency mode corresponds to an in-plane vibration, while the two higher-frequency peaks originate from out-of-plane motions. Among the vibrational modes, the one at 137.1 cm^–1^ shows the strongest intensity. It corresponds to an out-of-plane vibration where the two sublayers move in opposite directions relative to each other, while Sn and Te atoms within each layer move coherently. This collective displacement strongly modulates the interlayer dipole moment, giving rise to pronounced polarization and Raman activity.

In the hexagonal phase (Figure 5e–f), the optical phonon branches extend above 175 cm^–1^, reflecting stronger interatomic interactions. The Raman spectrum displays two dominant peaks at 130.2 cm^–1^ and 190.5 cm^–1^. The higher-frequency mode arises from an out-of-plane vibration with opposite Sn–Te displacements along the *z* axis, producing strong bond polarization and yielding the most intense Raman signature of this phase.

The β′ phase (Figure 5g–h) exhibits the broadest distribution of optical modes, reaching up to ∼170 cm^–1^. Its Raman spectrum features four peaks at 42.1 cm^–1^, 46.2 cm^–1^, 118.1 cm^–1^, and 168.6 cm^–1^, providing the richest vibrational fingerprint among the phases shown. The most intense mode at 118.1 cm^–1^ corresponds to a strong in-plane vibration with opposing Sn–Te motions, generating pronounced bond polarization, while the higher-frequency mode at 168.6 cm^–1^ arises from an out-of-plane vibration with opposite Sn–Te displacements. These multiple, well-separated Raman features offer clear experimental markers for identifying the β′ phase.

### 3.3 Phase-Dependent Transport Properties of SnTe

To evaluate intrinsic carrier transport in phase-engineered SnTe monolayers, we estimated the acoustic-phonon-limited carrier mobilities of the cubic, γ, hexagonal, and β′ phases using the linear deformation-potential (DP) formalism originally developed by Bardeen and Shockley for longitudinal-acoustic (LA) phonon scattering.^51^ Within this framework, the mobility is controlled by the in-plane elastic stiffness (*C*_2D_), the band-edge effective masses, and the deformation potential constant (*E*_1_) that quantifies electron–phonon coupling under strain. Because the method neglects extrinsic scattering from defects, interfaces, grain or domain boundaries, and substrate-related scattering channels, the resulting values represent intrinsic upper bounds for defect-free crystals and provide a benchmark for interpreting experimental transport. Accordingly, these values should not be interpreted as direct predictions of device performance, which can be substantially reduced in real samples by defect scattering, interface roughness, grain or domain boundaries, and substrate-related effects.

The carrier mobility is expressed as

3 $$\mu =\frac{e\hbar^{3}C_{2D}}{k_{B}Tm_{d}\left|m^{*}\right|E_{1}^{2}}$$

where *C*_2D_ is the in-plane elastic modulus, m* is the carrier effective mass along the transport direction, *m*_*d*_ is the density-of-states effective mass, and *E*_1_ is the deformation potential constant. *e*, *ℏ*, *k*_*B*_, and *T* denote the elementary charge, reduced Planck constant, Boltzmann constant, and temperature (300 K), respectively.

To determine the elastic response, uniaxial strain in the range of −0.5% ≤ *ε* ≤ +0.5% was applied along the *x* and *y* directions. The γ and cubic phases retained their native rectangular cell, while the β′ and hexagonal phases were transformed into orthorhombic supercells to enable directional strain application (see Figure S5 in SI). The in-plane elastic modulus *C*_2D_ characterizes the mechanical rigidity of the monolayer against uniaxial deformation and is defined as

4 $$C_{2D}=\frac{1}{S_{0}}\frac{\partial^{2}E}{\partial \varepsilon^{2}}$$

where *S*_0_ is the equilibrium surface area and *ε* is the applied strain. In practice, *C*_2D_ is obtained from a quadratic fit of the total energy as a function of small uniaxial strains applied along the *x* and *y* directions (Figure S6). Because eq 3 scales linearly with *C*_2D_, stiffer lattices tend to support higher intrinsic mobilities when the electronic factors are comparable.^52^

The deformation potential constant *E*_1_ quantifies the sensitivity of the CBM (electrons) or VBM (holes) to strain,

5 $$E_{1}=\frac{dE_{\mathrm{CBM}/\mathrm{VBM}}}{d\varepsilon }$$

*E*_1_ was determined by tracking the strain-induced shift of the CBM or VBM as a function of applied uniaxial strain. Linear fits of the band-edge positions with respect to strain were then performed to extract the deformation potential constants (see Figure S7).

The transport effective mass is extracted from the band curvature near the extremum,

6 $$m^{*}=\hbar^{2}{\left(\frac{d^{2}E}{dk^{2}}\right)}^{-1}$$

For anisotropic systems, scattering and the phase space available near the band edge depend on the overall in-plane curvature, captured by the density-of-states effective mass

7 $$m_{d}=\sqrt{m_{x}^{*}m_{y}^{*}}$$

which is the geometric mean of the principal in-plane effective masses. A larger *m*_*d*_ increases the density of available states near the band edge and typically strengthens carrier–phonon scattering, which reduces mobility through the inverse dependence on *m*_*d*_|m*| in eq 3.

From an experimental perspective, eq 3 captures only LA-phonon-limited transport in the linear DP approximation. In real SnTe monolayers and thin films, measured mobilities are often governed by additional scattering channels such as ionized impurities, grain boundaries, interface roughness, substrate-induced remote phonons, and phase or domain boundaries in low-symmetry polymorphs.^26,53^ Accordingly, the DP mobilities reported here should be interpreted as intrinsic benchmarks that isolate the lattice- and band-structure-limited contribution, enabling a quantitative estimate of the extrinsic scattering budget in experiments.

Table 2 reveals a pronounced dependence of *C*_2D_ and *E*_1_ on crystal phase. The cubic phase is significantly stiffer than the other polymorphs, with *C*_2D_ ≈ 124 N/m along both *x* and *y*, indicating a rigid bonding network against in-plane deformation. By contrast, the γ, hexagonal, and β′ phases are mechanically softer, with *C*_2D_ in the ∼29–42 N/m range, consistent with a more compliant lattice response. The deformation potentials are also phase and direction dependent. The hexagonal phase exhibits a particularly small electron DP constant along *x* $\left(E_{1}^{e}=1.045\;\mathrm{eV}\right)$ , whereas γ-SnTe shows comparatively large electron DP constants $\left(E_{1}^{e}∼5.15-5.40\;\mathrm{eV}\right)$ , which suppress mobility through the $E_{1}^{-2}$ dependence in eq 3.

**Table 2:** Deformation Potential Constant of Electrons and Holes (*E*_1_), Elastic Constant (*C*_2*D*_), Effective Mass of Electron and Hole $\left(m_{e}^{*},m_{h}^{*}\right)$ , and Carrier Mobility of Electron and Hole (μ_*e*_, μ_*h*_) at 300 K in the *X* and *Y* Directions for Monolayer SnTe in the Cubic, *γ*, Hexagonal, and *β*′ Phases

Phase	Dir.	$E_{1}^{e}\;\left(\mathrm{eV}\right)$	$E_{1}^{h}\;\left(\mathrm{eV}\right)$	*C*_2D_ (N/m)	$m_{e}^{*}\;\left(m_{0}\right)$	$m_{h}^{*}\;\left(m_{0}\right)$	μ_*e*_ (×10^3^ cm^2^/(V s))	μ_*h*_ (×10^3^ cm^2^/(V s))
Cubic	x	3.08	9.19	124.26	0.94	0.04	0.98	6.29
	y	3.08	9.19	124.26	0.94	0.04	0.98	6.29
γ	x	5.15	8.01	37.35	0.19	0.16	1.88	0.70
	y	5.40	8.78	41.71	0.11	0.12	1.91	0.65
Hexagonal	x	1.045	8.30	29.62	0.54	0.62	6.02	0.02
	y	4.13	8.50	29.58	0.26	0.68	0.38	0.01
β′	x	2.03	1.74	38.68	0.57	0.53	0.91	0.93
	y	5.03	1.36	38.72	0.44	0.54	0.14	1.53

Despite the large stiffness, electron transport in cubic SnTe remains modest because the electron effective mass is heavy $\left(m_{e}^{*}\approx 0.94\;m_{0}\right)$ , yielding μ_*e*_ ≈ 980 cm^2^/(V s) in both directions. In sharp contrast, the VBM is highly dispersive, producing an ultralight hole mass $\left(m_{h}^{*}\approx 0.04\;m_{0}\right)$ and a correspondingly high intrinsic hole mobility of μ_*h*_ ≈ 6.29 × 10^3^ cm^2^/(V s). This large μ_*h*_ persists even though the hole deformation potential is sizable $\left(E_{1}^{h}=9.19\;\mathrm{eV}\right)$ , indicating that, in the cubic phase, the unusually small $m_{h}^{*}$ dominates the intrinsic hole-transport limit. This combination suggests that high-quality, strain-relaxed cubic SnTe is a favorable platform for hole-dominated devices, provided that defect- and interface-related scattering are minimized.^23,25^

The γ phase exhibits substantially lighter electron masses ( $m_{e}^{*}\approx 0.19{\;m}_{0}$ along *x* and 0.11 *m*_0_ along *y*), which boosts electron mobility to μ_*e*_ ≈ 1880–1910 cm^2^/(V s). Compared with the cubic phase, the softer lattice (*C*_2D_ ≈ 37–42 N/m) and the relatively large electron deformation potentials $\left(E_{1}^{e}∼5.15-5.40\;\mathrm{eV}\right)$ limit further enhancement. Hole mobilities remain moderate, μ_*h*_ ≈ (0.65–0.70) × 10^3^ cm^2^/(V s), reflecting the combined influence of moderate hole masses $\left(m_{h}^{*}\approx 0.12-0.16\;m_{0}\right)$ and sizable hole deformation potentials $\left(E_{1}^{h}∼8.01-8.78\;\mathrm{eV}\right)$ .

The hexagonal phase displays pronounced anisotropy as shown in Figure 6. Along *x*, the small electron deformation potential $\left(E_{1}^{e}=1.045\;\mathrm{eV}\right)$ yields a high electron mobility of μ_*e*_ ≈ 6020 cm^2^/(V s). Along *y*, the electron DP constant increases to $E_{1}^{e}=4.13\;\mathrm{eV}$ , and the electron mobility drops to μ_*e*_ ≈ 38 cm^2^/(V s), despite a similar *C*_2D_. Hole transport is strongly hindered in both directions, with μ_*h*_ ≈ 10–20 cm^2^/(V s), consistent with heavy hole masses $\left(m_{h}^{*}\approx 0.62-0.68\;m_{0}\right)$ and large $E_{1}^{h}\left(∼8.30-8.50\;\mathrm{eV}\right)$ . This combination indicates that, if the hexagonal phase is realized experimentally, it is most promising for directionally engineered electron conduction along the high-mobility axis, while *p*-type transport would be intrinsically limited even before extrinsic scattering is considered.

**Figure 6:**
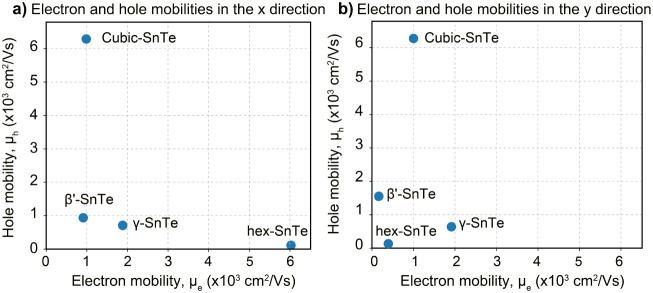
(a) Electron (μ*_e_*) and (b) hole (μ*_h_*) mobilities of cubic, γ, hexagonal, and β′-SnTe along the *x* and *y* directions.

The β′ phase also exhibits strong direction dependence (Figure 6). Along *x*, the electron mobility is μ_*e*_ ≈ 910 cm^2^/(V s), while along *y* it decreases to μ_*e*_ ≈ 140 cm^2^/(V s). This suppression is consistent with the larger electron deformation potential along *y* $\left(E_{1}^{e}=5.03\;\mathrm{eV}\right)$ relative to *x* $\left(E_{1}^{e}=2.03\;\mathrm{eV}\right)$ . In contrast, hole mobility is comparatively high and anisotropic: μ_*h*_ ≈ 0.93 × 10^3^ cm^2^/(V s) along *x* and increases to μ_*h*_ ≈ 1.53 × 10^3^ cm^2^/(V s) along *y*. This trend follows the small hole deformation potentials in β′-SnTe ( $E_{1}^{h}=1.74\;\mathrm{eV}$ along *x* and 1.36 eV along *y*), which enhance mobility through the $E_{1}^{-2}$ dependence in eq 3. The β′ phase therefore emerges as a promising candidate for high-mobility hole transport in selected in-plane orientations, which is particularly relevant given the common *p*-type character of SnTe.

Experimental studies on bulk and thin-film SnTe with the cubic structure report a broad range of mobilities, strongly influenced by temperature, carrier concentration, and growth conditions.^22^ High mobilities have been achieved in epitaxial thin films; SnTe grown on Bi_2_Te_3_ buffer layers exhibits mobilities of approximately 2000 cm^2^/(V s) at 15 K,^25^ while SnTe/CdTe heterostructures show values of about 992 cm^2^/(V s) at 4 K.^23^ In contrast, polycrystalline samples and room-temperature measurements typically yield substantially reduced mobilities, often below 100 cm^2^/(V s).^26^ Within this context, our DP-limited values at 300 K provide a phase-resolved intrinsic baseline. For cubic SnTe, μ_*e*_ ≈ 10^2^ cm^2^/(V s) and μ_*h*_ reaches the 10^3^ cm^2^/(V s) scale, indicating that room-temperature transport in high-quality films can still be substantially reduced by extrinsic mechanisms, particularly for holes. For the γ, hexagonal, and β′ phases, the computed mobilities fall in experimentally accessible ranges but exhibit strong orientation dependence, suggesting that domain texture and phase purity will directly impact measured in-plane anisotropy.

The phase-resolved trends in Table 2 suggest several actionable strategies for experiments. First, strain control is critical. Because $\mu \propto E_{1}^{-2}$ in eq 3, reducing residual strain and strain gradients through substrate selection, thermal-expansion matching, and postgrowth relaxation can strongly improve mobility, particularly in phases or directions with large *E*_1_.^52^ Second, in-plane anisotropy can serve as an electrical fingerprint of phase texture and domain alignment. The large predicted contrast for hexagonal SnTe between *x* and *y* implies that angle-resolved transport measurements using rotated Hall bars or multiterminal geometries can help identify domain orientation, complementing Raman- and diffraction-based phase assignment.^26^ Third, the DP limits enable quantitative benchmarking of extrinsic scattering. Since SnTe is often *p*-type due to native acceptors,^53^ comparison of measured μ_*h*_ against the intrinsic values in Table 2 provides a direct estimate of mobility suppression from defects, interfaces, and phase or domain boundaries, thereby guiding growth and processing strategies.

Overall, the intrinsic mobility of SnTe monolayers is strongly phase dependent and arises from the interplay of lattice rigidity (*C*_2D_), band-edge curvature (effective masses), and deformation-potential coupling strength (*E*_1_). As shown in Figure 6 the cubic phase supports very high DP-limited hole transport due to its exceptionally light valence-band curvature, γ-SnTe provides comparatively balanced electron mobility with moderate holes, hexagonal SnTe enables highly anisotropic electron conduction along the favorable in-plane axis while suppressing hole transport, and β′-SnTe supports direction-selective high-mobility holes enabled by small hole deformation potentials. These phase-specific signatures provide an experimentally testable map for using structural phase engineering to tailor in-plane transport in two-dimensional SnTe.

### 3.4 Substitutional Doping in Cubic- and γ-SnTe

To investigate substitutional doping in cubic SnTe, a 2 × 2 × 1 supercell was constructed from the optimized primitive structure. Within this supercell, the Sn sublattice was categorized into four symmetry-inequivalent substitutional sites, as illustrated in Figure 2. For each dopant species (Bi, Sb, Mn, Ge, Cr, V, and Co), one Sn atom at each inequivalent site was replaced, generating four distinct candidate configurations. Following full structural relaxation, the total energies of these configurations were evaluated within both spin-unpolarized (ISPIN = 1) and spin-polarized (ISPIN = 2) formalisms. The energetically preferred configurations were found to correspond to site 1 for Bi and Sb, site 3 for Mn, Cr, and V, and site 4 for Ge and Co (Table S1 in the SI). It should be noted that, for some dopants, two substitutional sites were found to be nearly degenerate in total energy and exhibited very similar magnetic behavior. In such cases, one of these nearly equivalent configurations was selected as the representative structure for the subsequent analysis, based on the lowest calculated total energy (see Table S1). These lowest-energy geometries were subsequently adopted for the calculation of formation energies, magnetic moments, and unfolded electronic band structures.

An analogous procedure was applied to the γ-SnTe phase. In this case, a 3 × 3 × 1 supercell was generated from the optimized primitive cell, and the Sn sublattice was resolved into two symmetry-inequivalent substitutional sites, as shown in Figure 3. For each dopant, substitution at both inequivalent Sn sites was considered, producing two candidate configurations. After structural optimization and total-energy comparison within both nonspin-polarized and spin-polarized schemes, the most stable configurations were identified as site 1 for Bi, Mn, and Co, and site 2 for Sb, Ge, Cr, and V (see Table S2). These energetically favorable structures were then used in the subsequent analysis of thermodynamic stability, magnetic response, and electronic properties.

The thermodynamic stability of substitutional doping was evaluated through the defect formation energy. For a dopant atom *X* substituting a Sn atom, the formation energy was calculated according to^54^

8 $$E_{f}\left(\mathrm{doped}\right)=E_{\mathrm{tot}}\left(\mathrm{doped}\right)-E_{\mathrm{tot}}\left(\mathrm{pristine}\right)-\mu_{X}+\mu_{\mathrm{Sn}}$$

where *E*_tot_(doped) and *E*_tot_(pristine) denote the total energies of the doped and pristine supercells, respectively, while μ_*X*_ and μ_Sn_ represent the chemical potentials of the dopant atom and the substituted Sn atom. In these substitutional-doping calculations, the chemical potentials of the removed Sn atom and the added dopant atom were referenced to their elemental bulk phases when evaluating the formation energies.

Because the doped systems were modeled using supercells, the corresponding electronic bands are folded into the smaller Brillouin zones of the enlarged cells, producing a dense manifold of states that obscures the physically meaningful host-band dispersion.^55^ To recover the effective electronic structure in the primitive Brillouin zone and enable a direct comparison with the pristine phases, band-unfolding calculations were performed along the high-symmetry paths of the corresponding primitive cells. The resulting unfolded band structures for doped cubic and γ-SnTe are presented in Figure S1a and b in the SI.

For cubic SnTe, the calculated defect formation energies in Table 3 show a clear dopant dependence, with Mn substitution being the most energetically favorable among all considered impurities. Here, *M* denotes the total magnetic moment of the defective supercell. Its formation energy of 0.36 eV/atom is lower than those of Bi, Sb, Ge, Cr, V, and Co, indicating that Mn incorporation is thermodynamically preferred in the cubic phase. This favorable energetic stability is accompanied by a pronounced magnetic response, as Mn induces a large magnetic moment of 4.47 μ_B_, in clear contrast to the weakly magnetic Bi- and Sb-substituted systems and the nonmagnetic Ge case. The combination of low formation energy and strong spin polarization therefore identifies Mn as the most effective dopant for introducing magnetism into cubic SnTe.

**Table 3:** Calculated Defect Formation Energy *E_f_*, Total Magnetic Moment of the Defective Supercell *M*, and Band Gap *E_g_* of Doped Cubic-SnTe and *γ*-SnTe

Phase	Dopant	*E*_*f*_ (eV/atom)	*M* (μ_B_)	*E*_*g*_ (eV)
Cubic-SnTe	Bi	0.60	0.61	Metal
	Sb	0.90	0.49	Metal
	Mn	0.36	4.47	Metal
	Ge	0.49	NM	Metal
	Cr	1.17	3.67	Metal
	V	1.14	2.68	Metal
	Co	1.73	2.55	Metal
γ-SnTe	Bi	0.96	NM	Metal
	Sb	1.24	NM	Metal
	Mn	0.38	4.43	0.55 (Direct)
	Ge	0.49	NM	0.49 (Indirect), 0.50 (Direct)
	Cr	1.29	3.62	0.23 (Indirect), 0.32 (Direct)
	V	1.40	2.68	Metal
	Co	1.81	2.52	0.14 (Direct)

The unfolded electronic band structures in Figure 7 further show that substitutional doping substantially modifies the low-energy electronic states of cubic SnTe. In pristine cubic SnTe, the near-Fermi-level dispersion retains the characteristic Dirac-like feature around the M point. Upon doping, however, this feature is systematically shifted to lower energy, falling below the Fermi level in all cases considered. This downward displacement produces finite spectral weight at the Fermi level and gives rise to an overall metallic character, consistent with the band gap values listed in Table 3. From an electronic-transport perspective, this behavior indicates an electron-doping tendency, i.e., an n-type shift of the Fermi level relative to the host band structure.

**Figure 7:**
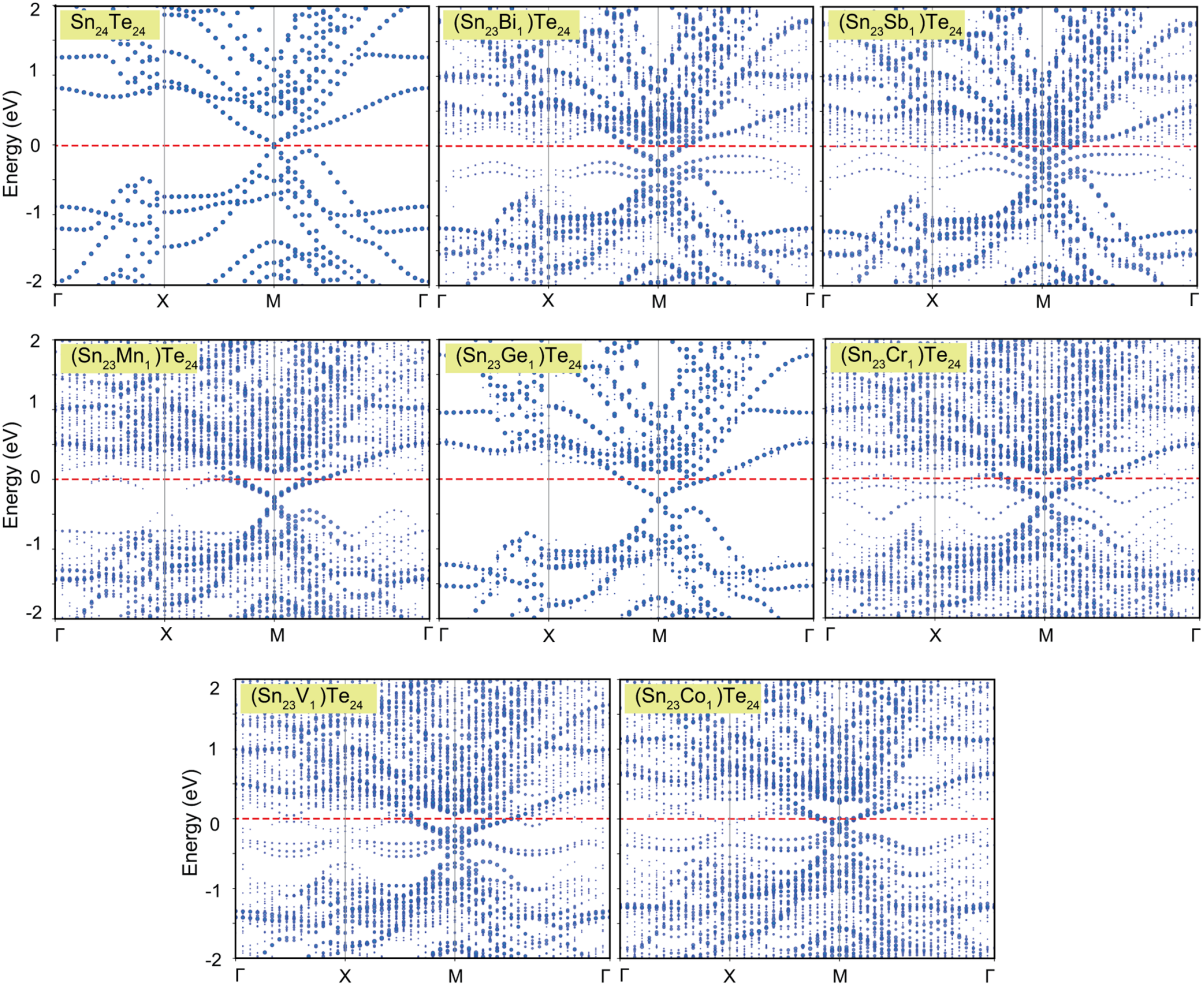
Unfolded electronic band structures of pristine and doped cubic SnTe calculated using the GGA+SOC method along the high-symmetry path Γ–X–M−Γ. The red dashed line indicates the Fermi level, which is set to 0 eV.

Although all dopants drive cubic SnTe toward metallic behavior, the extent to which they perturb the host dispersion differs significantly. Ge substitution, which has the second-lowest formation energy after Mn, introduces only a comparatively moderate reconstruction of the band structure. The overall dispersion of the host phase remains largely recognizable, despite the downward shift of the Dirac-like feature, and no magnetic moment is induced. This suggests that Ge acts as a relatively gentle electronic perturbation, favoring n-type metallicity without activating spin polarization. By contrast, Mn not only shifts the low-energy states below the Fermi level but also produces a much stronger redistribution of spectral weight in the vicinity of the crossing region, reflecting the combined effects of symmetry breaking and spin polarization introduced by the transition-metal dopant.

A similar but dopant-dependent trend is also observed for Bi and Sb substitution. These two main-group dopants remain reasonably accessible energetically, with formation energies of 0.60 and 0.90 eV/atom, respectively, and they preserve the overall shape of the host band dispersion more effectively than the transition-metal dopants. Nevertheless, both induce a finite magnetic moment, albeit small, of 0.61 and 0.49 μ_B_, respectively, indicating weak spin polarization. In contrast, Cr, V, and Co exhibit larger formation energies and stronger band-structure perturbations, particularly near the Fermi level, where the unfolded spectra become more diffuse and distorted. Among these, Cr induces a relatively large magnetic moment of 3.67 μ_B_, while V and Co also generate sizable moments of 2.68 and 2.55 μ_B_, respectively. However, their higher formation energies suggest that these dopants are less favorable from a thermodynamic standpoint than Mn.

For γ-SnTe, the defect formation energies listed in Table 3 again reveal a clear dopant preference, with Mn substitution being the most energetically favorable among all considered impurities. Its formation energy of 0.38 eV/atom is substantially lower than those of Bi, Sb, Cr, V, and Co, and is also slightly lower than that of Ge, indicating that Mn is the most favorable dopant for incorporation into the γ phase. Importantly, this energetic preference is accompanied by a strong magnetic response, with Mn inducing a magnetic moment of 4.43 μ_B_, which is very close to the value obtained for Mn-doped cubic SnTe. Unlike the cubic case, however, Mn substitution in γ-SnTe preserves the semiconducting character of the host phase and yields a direct band gap of 0.55 eV. This combination of low formation energy, robust spin polarization, and retained semiconducting behavior makes Mn particularly attractive for realizing magnetic semiconducting γ-SnTe.

The unfolded band structures in Figure 8 show that Mn doping modifies the low-energy states of γ-SnTe without destroying the overall band topology of the host phase. Although some band broadening and spectral redistribution are visible due to the broken translational symmetry introduced by substitution, the main features of pristine γ-SnTe remain identifiable. At the same time, the low-energy electronic states are shifted slightly toward lower energies, indicating electron donation from the dopant and an n-type tendency. In contrast to cubic SnTe, where all dopants drive the system into a metallic state, the γ phase exhibits a more diverse response, with both metallic and semiconducting outcomes depending on the dopant species.

**Figure 8:**
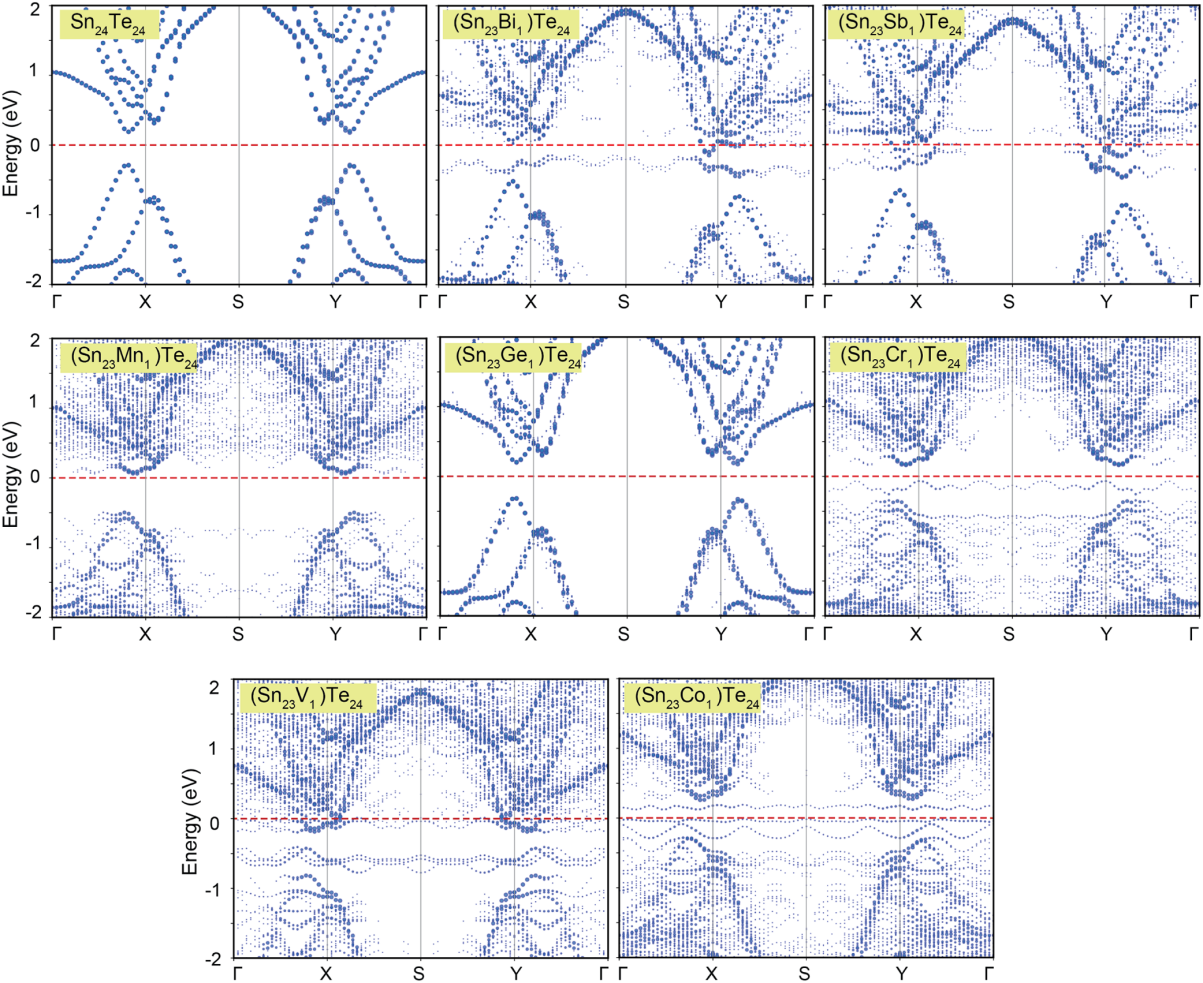
Unfolded electronic band structures of pristine and doped γ-SnTe calculated using the GGA+SOC method along the high-symmetry path Γ–X–S–Y−Γ. The red dashed line indicates the Fermi level, which is set to 0 eV.

Ge substitution shows the second-lowest formation energy, 0.49 eV/atom, and represents the most favorable nonmagnetic dopant in the γ phase. As seen in Figure 8, Ge causes only limited perturbation to the host electronic structure, and the overall dispersion remains close to that of pristine γ-SnTe. Consistent with this weak perturbation, Ge-doped γ-SnTe remains semiconducting, with an indirect band gap of 0.49 eV and a nearly identical direct gap of 0.50 eV. Thus, Ge provides a relatively gentle route for modifying the electronic structure while preserving both the semiconducting character and the nonmagnetic nature of the host material.

A different behavior is found for Bi and Sb substitution. Although these main-group dopants do not induce magnetism in γ-SnTe, with both systems remaining nonmagnetic, they shift the low-energy states below the Fermi level sufficiently to generate metallic behavior. Their formation energies, 0.96 and 1.24 eV/atom for Bi and Sb, respectively, are notably larger than those of Mn and Ge, indicating that they are less favorable energetically. Nevertheless, the corresponding unfolded band structures suggest an electron-doping effect similar to that observed in the cubic phase, again reflecting an n-type character associated with the downward displacement of the near-edge states.

Among the transition-metal dopants beyond Mn, Cr, V, and Co all introduce substantial spin polarization, with magnetic moments of 3.62, 2.68, and 2.52 μ_B_, respectively. Their electronic effects, however, are more diverse. Cr-doped γ-SnTe remains semiconducting, with an indirect band gap of 0.23 eV and a direct band gap of 0.32 eV, while Co doping yields a narrow direct-gap semiconductor with *E*_*g*_ = 0.14 eV. In both cases, the unfolded spectra show stronger distortion and band broadening than in the Ge- or Mn-doped systems, indicating a more pronounced reconstruction of the host electronic states. By contrast, V doping drives the system metallic, despite also inducing a sizable magnetic moment. Therefore, while Cr, V, and Co all produce spin-polarized states, only Cr and Co preserve semiconducting behavior in the γ phase.

In contrast to cubic SnTe, where substitution universally drives the system toward metallicity, γ-SnTe exhibits a richer dopant-dependent electronic response in which magnetic, metallic, and semiconducting states can all be selectively accessed. This enhanced flexibility is particularly evident for Mn doping, which uniquely combines thermodynamic favorability, strong spin polarization, and preserved semiconducting character. The γ phase therefore appears especially promising as a defect-engineerable SnTe polymorph for achieving magnetic semiconducting functionality within a single 2D material platform.

### 3.5 Vacancy Defects in Cubic- and γ-SnTe

Vacancy-defective models were constructed for both cubic- and γ-SnTe by removing one Sn atom or one Te atom from the corresponding supercells. For cubic SnTe, a 2 × 2 × 1 supercell was used, and both the Sn and Te sublattices were classified into four symmetry-inequivalent site groups, as shown in Figure 2. One atom was removed from each site group to generate candidate configurations for V_Sn_ and V_Te_. Total energies were then compared after full structural relaxation using both spin-unpolarized (ISPIN = 1) and spin-polarized (ISPIN = 2) calculations. The energetically most favorable configurations were identified as site 1 for V_Sn_ and site 4 for V_Te_, according to Table S3. For γ-SnTe, a 3 × 3 × 1 supercell was employed, in which the Sn and Te sublattices each contain two symmetry-inequivalent site groups, as illustrated in Figure 3. Following the same procedure, the most favorable vacancy configurations were determined to be site 2 for V_Sn_ and site 1 for V_Te_, as summarized in Table S4. These lowest-energy vacancy structures were subsequently used for the calculation of defect formation energies, magnetic moments, and unfolded electronic band structures.

The vacancy formation energy was evaluated using

9 $$E_{f}\left(V_{X}\right)=E_{\mathrm{tot}}\left(V_{X}\right)-E_{\mathrm{tot}}\left(\mathrm{pristine}\right)+\mu_{X}$$

where *E*_tot_(V_*X*_) and *E*_tot_(pristine) are the total energies of the defective and pristine supercells, respectively, and μ_*X*_ is the chemical potential of the removed atom *X* (*X* = Sn or Te). This quantity represents the energetic cost of creating a vacancy and provides a measure of the relative tendency for vacancy formation.

For cubic SnTe, both V_Sn_ and V_Te_ preserve metallic behavior, as indicated by the finite spectral weight at the Fermi level in the unfolded band structures and the absence of a band gap in Table 4. However, the two vacancy types affect the near-Fermi-level dispersion in different ways (see Figure 9). In the V_Te_ case, the Dirac-like crossing shifts downward with respect to the Fermi level, whereas in the V_Sn_ case it shifts upward. These opposite trends indicate that Te and Sn vacancies perturb the carrier balance differently, even though both defective cubic systems remain metallic. Compared with pristine cubic SnTe, both vacancies also introduce substantial band broadening and noticeable redistribution of spectral weight around the Fermi level due to the broken periodicity and the local perturbation created by atomic removal. Among the two vacancy types, V_Sn_ is energetically more favorable, with a formation energy of 0.87 eV/atom, whereas V_Te_ has a significantly higher formation energy of 1.40 eV/atom. This energetic ordering indicates that Sn vacancies are more readily formed than Te vacancies in the cubic phase. In both cases, the calculated magnetic moment remains zero, showing that vacancy formation in cubic SnTe does not induce stable spin polarization within the present computational framework.

**Table 4:** Calculated Defect Formation Energy *E_f_*, Total Magnetic Moment of the Defective Supercell *M*, and Band Gap *E_g_* of Sn and Te Vacancies in Cubic-SnTe and *γ*-SnTe

Phase	Defect	*E*_*f*_ (eV/atom)	*M* (μ_B_)	*E*_*g*_ (eV)
Cubic-SnTe	V_Sn_	0.87	NM	Metal
	V_Te_	1.40	NM	Metal
γ-SnTe	V_Sn_	0.99	NM	Metal
	V_Te_	1.52	NM	Metal

**Figure 9:**
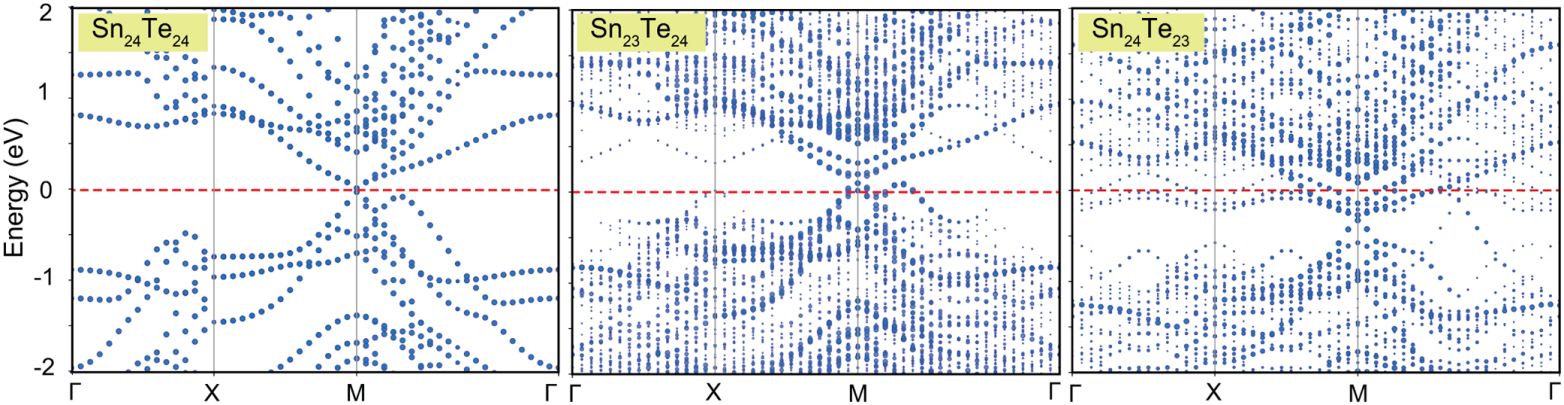
Unfolded electronic band structures of pristine and vacancy-defective cubic SnTe calculated using the GGA+SOC method along the high-symmetry path Γ–X–M−Γ. The red dashed line indicates the Fermi level, which is set to 0 eV.

A similar but electronically more explicit trend is obtained for γ-SnTe. Although both V_Sn_ and V_Te_ exhibit metallic character, the unfolded band structures indicate different carrier-type tendencies (see Figure 10). The Te vacancy introduces donor-like behavior and drives the system toward an n-type electronic character, whereas the Sn vacancy produces acceptor-like behavior and leads to p-type character. Relative to pristine γ-SnTe, both vacancy-defective systems show clear broadening and distortion of the host bands, particularly in the near-Fermi-level region, reflecting the perturbation introduced by the missing atom. As in the cubic phase, V_Sn_ is thermodynamically more favorable than V_Te_, with formation energies of 0.99 and 1.52 eV/atom, respectively. Both vacancy types remain nonmagnetic, indicating that neither Sn nor Te vacancies generate a stable local magnetic moment in γ-SnTe.

**Figure 10:**
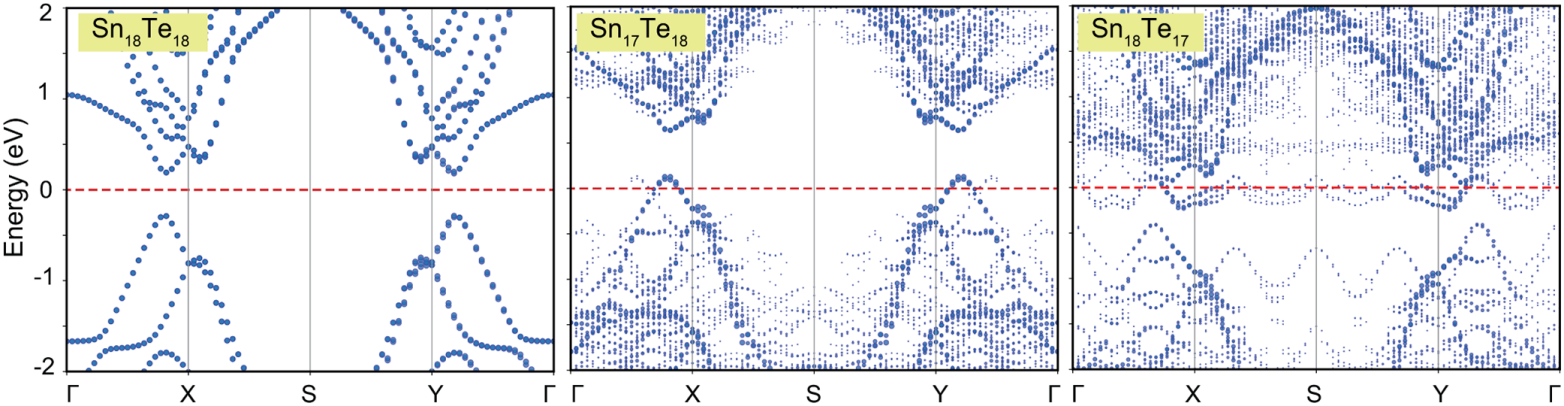
Unfolded electronic band structures of pristine and vacancy-defective γ-SnTe calculated using the GGA+SOC method along the high-symmetry path Γ–X–S–Y−Γ. The red dashed line indicates the Fermi level, which is set to 0 eV.

Overall, the vacancy results reveal a consistent trend across both SnTe phases. Sn vacancies are energetically more favorable than Te vacancies, suggesting that they are the more probable intrinsic point defects under comparable conditions. This is also consistent with experimental reports showing that pristine SnTe is typically p-type due to the presence of intrinsic Sn vacancies.^56,57^ At the electronic-structure level, the vacancy and substitution results suggest a particularly appealing defect-engineering route in monolayer SnTe. Substitutional doping in both cubic and γ-SnTe generally drives the system toward donor-like, *n*-type behavior, whereas Sn vacancies favor acceptor-like, *p*-type character. This contrast indicates that opposite carrier polarities may be achievable within the same SnTe material family through selective defect engineering alone. Such behavior is especially attractive for the design of homomaterial *p*–*n* junctions, where *n*-type regions could be realized by appropriate dopant incorporation and *p*-type regions by Sn-deficient growth conditions. In principle, this opens a pathway toward SnTe-based junction architectures for optoelectronic applications, including photodetectors and gate-tunable photoresponsive devices, without requiring heterogeneous material integration.

From a defect-engineering perspective, intrinsic vacancies play a complementary role to substitutional dopants in monolayer SnTe. In both cubic- and γ-SnTe, Sn vacancies are energetically more favorable than Te vacancies and consistently give rise to acceptor-like behavior, whereas substitutional dopants more often drive donor-like or metallic responses. This contrast is important because it shows that native vacancies are not merely secondary defects, but can provide a distinct and experimentally relevant route for tuning carrier polarity in monolayer SnTe. Since such vacancies are often unavoidable during growth, their effects should be considered alongside those of deliberate chemical substitution when evaluating the functional defect landscape of this material platform.^2,36,37^

It is also worth noting that some substitutional-doping and vacancy configurations have very similar formation energies (Tables 3 and 4). In particular, the formation energy of Sb-substituted cubic SnTe (0.90 eV/atom) is very close to that of a Sn vacancy in cubic SnTe (0.87 eV/atom), while in γ-SnTe the formation energy of Bi substitution (0.96 eV/atom) is similarly close to that of a Sn vacancy (0.99 eV/atom). This near-degeneracy suggests that, under suitable growth conditions, incorporation of Sb in cubic SnTe or Bi in γ-SnTe may compete with native Sn-vacancy formation. From an experimental perspective, such behavior may complicate the selective realization of purely doped configurations and indicates that Bi/Sb incorporation should be considered together with the possible presence of Sn vacancies when interpreting synthesis outcomes and carrier-type trends.

## 4 Conclusions

In conclusion, our results show that monolayer SnTe possesses a distinctly phase-dependent structure–property relationship, in which crystal polymorphism first defines the intrinsic energetic and functional landscape of the material. Among the four examined polymorphs, cubic SnTe is the most stable monolayer with *E*_coh_ = 6.63 eV/f.u. and *E*_for_ = −0.53 eV/f.u., closely followed by γ-SnTe with *E*_coh_ = 6.59 eV/f.u. and *E*_for_ = −0.49 eV/f.u., while the hexagonal and β′ phases are less favorable. This structural competition is directly reflected in the electronic response: the HSE+SOC band gap ranges from 0.35 eV in cubic SnTe to 0.82 eV in γ-SnTe, 0.80 eV in β′-SnTe, and 1.84 eV in the hexagonal phase. The same phase dependence also governs intrinsic transport, yielding ultrahigh hole mobility of 6.29 × 10^3^ cm^2^ V^–1^ s^–1^ in cubic SnTe, comparatively balanced electron transport in γ-SnTe with μ_*e*_ ≈ 1.88–1.91 × 10^3^ cm^2^ V^–1^ s^–1^, strongly anisotropic electron transport in hexagonal SnTe reaching 6.02 × 10^3^ cm^2^ V^–1^ s^–1^ along the favorable direction, and anisotropic hole transport in β′-SnTe up to 1.53 × 10^3^ cm^2^ V^–1^ s^–1^. Taken together, these results establish that phase selection in monolayer SnTe is not merely a structural issue, but the primary factor controlling the accessible gap landscape and transport regime.

Building on this intrinsic phase dependence, defect engineering introduces a second and more selective level of control. In cubic SnTe, all considered substitutional dopants drive the system toward metallicity, showing that the near-Fermi-level states of this phase are highly sensitive to chemical substitution. In contrast, γ-SnTe responds in a much more diverse way, supporting metallic, narrow-gap, and semiconducting states depending on the dopant species. Within this defect landscape, Mn stands out as the most favorable and magnetically effective dopant in both phases, with formation energies of 0.36 eV/atom in cubic and 0.38 eV/atom in γ-SnTe, and magnetic moments of 4.47 and 4.43 μ_B_, respectively. Most importantly, Mn-doped γ-SnTe retains a direct band gap of 0.55 eV, making it the most promising candidate for magnetic semiconducting functionality in this system. However, the present single-dopant calculations do not by themselves establish long-range magnetic ordering; future work should therefore examine dopant–dopant exchange interactions, magnetic anisotropy, and finite-temperature magnetic behavior. Vacancy defects further complement this picture: Sn vacancies are more favorable than Te vacancies in both cubic (0.87 vs 1.40 eV/atom) and γ-SnTe (0.99 vs 1.52 eV/atom), remain nonmagnetic, and favor acceptor-like *p*-type behavior, whereas substitutional doping generally promotes donor-like *n*-type character.

Overall, the combined phase and defect results reveal a coherent design picture for monolayer SnTe. Phase selection primarily determines the intrinsic transport character and band gap range, while local chemical modification governs metallicity, magnetic response, and carrier polarity. These phase- and defect-dependent trends suggest distinct application directions within monolayer SnTe, with cubic- and γ-SnTe being relevant for infrared optoelectronic applications, hexagonal- and β′-SnTe for direction-sensitive transport devices, and Mn-doped γ-SnTe for spin-functional semiconducting applications. Together with the distinct Raman and STM fingerprints obtained for each polymorph, this establishes monolayer SnTe as a structurally identifiable and functionally designable two-dimensional material platform for infrared optoelectronics, anisotropic transport devices, and magnetic semiconducting applications.

## Supplementary Material



## Notes

Additional figures and tables, AIMD results, unfolded band structures, and strain-dependent transport analysis (PDF)
