# Supplementary material for: Phase-Dependent Functionality and Defect-Induced Magnetism in Monolayer SnTe Polymorphs
Source: ACS Appl Mater Interfaces. 2026 Jun 3;18(23):32903–20. doi: 10.1021/acsami.6c07072 (PMC13288409; doi:10.1021/acsami.6c07072)
Supplement: Supplementary file 1 [file am6c07072_si_001.pdf]

Supporting Information: Phase-Dependent Functionality and Defect-Induced Magnetism in Monolayer SnTe Polymorphs

Roya Kavkhani,[†,‖] Berna Akgenc Hanedar,[‡,‖] and Mehmet Cengiz Onbaşlı[*,†,¶,§]

†*Graduate School of Sciences and Engineering (GSSE), Koç University, Rumelifeneri Yolu, Sarıyer 34450, Istanbul, Türkiye*

‡*Department of Physics, Kırklareli University, 39100, Kırklareli, Türkiye*

¶*Department of Electrical & Electronics Engineering, Koç University, Rumelifeneri Yolu, Sarıyer 34450, Istanbul, Türkiye*

§*Department of Physics, Koç University, Rumelifeneri Yolu, Sarıyer 34450, Istanbul, Türkiye*

‖*These authors contributed equally to this work.*

E-mail: monbasli@ku.edu.tr

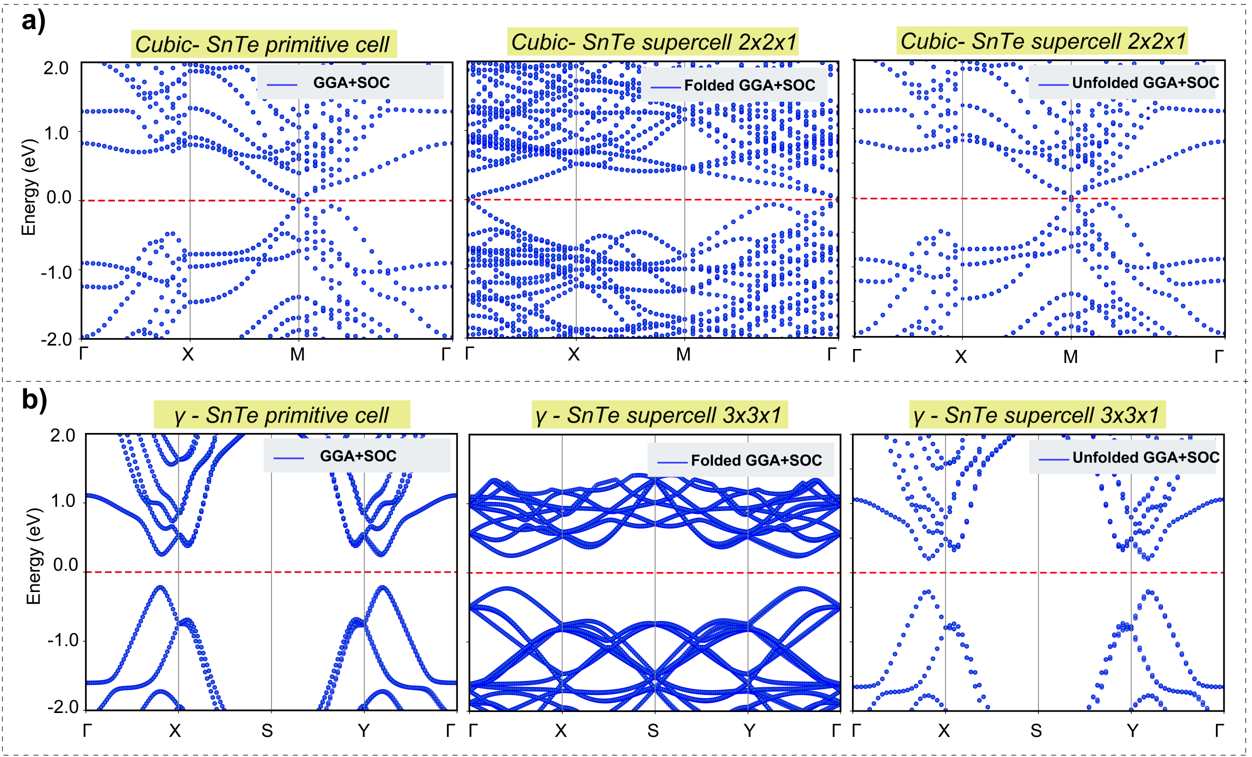

Figure S1. Electronic band structures of (a) cubic SnTe and (b) γ-SnTe calculated using the GGA+SOC method. The left panels show the band structure of the primitive cell, while the middle panels present the folded band structure obtained from the corresponding supercells ($2\times2\times1$ for cubic SnTe and $3\times3\times1$ for γ-SnTe). The right panels display the unfolded band structures projected onto the primitive Brillouin zone. The red dashed line indicates the Fermi level, which is set to 0 eV.

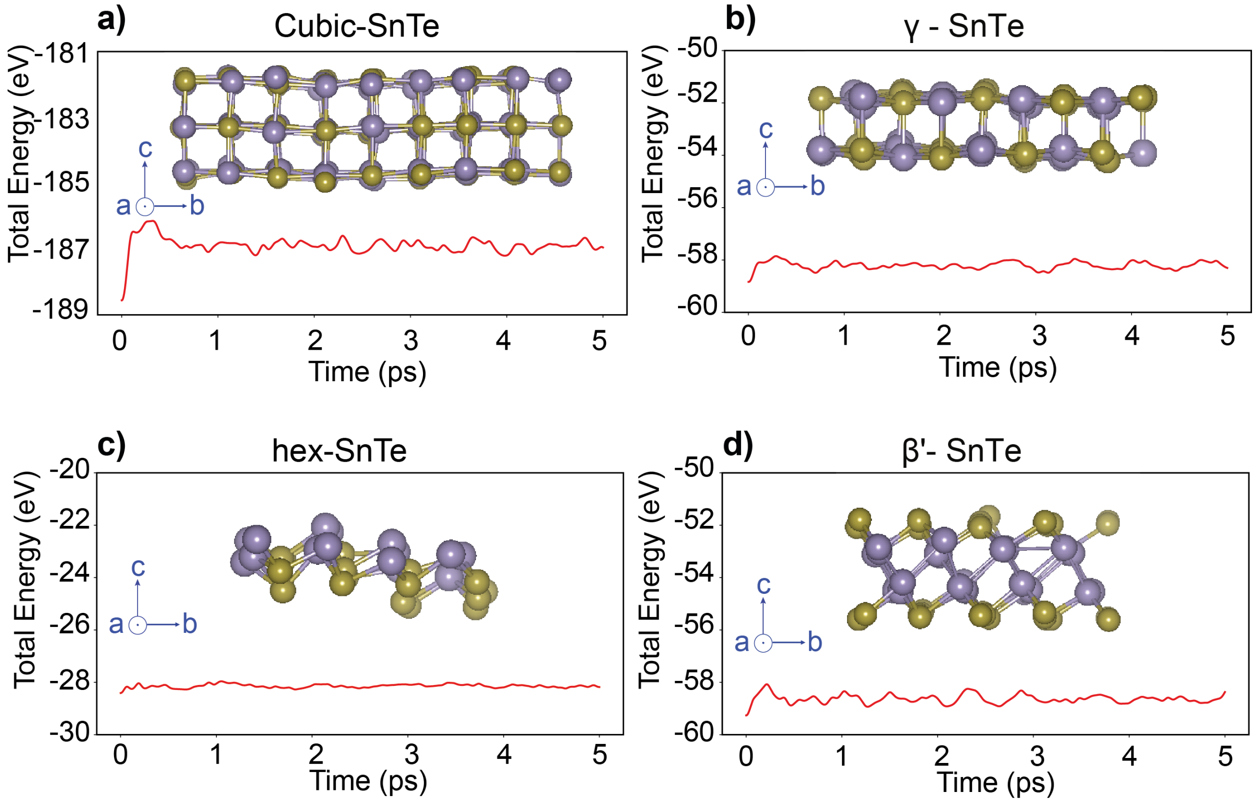

Figure S2. AIMD total-energy evolution (red) over 5 ps for pristine SnTe polymorphs; (a) cubic, (b) γ, (c) hex, and (d) β'. Insets show representative snapshots with lattice directions indicated; purple and gold spheres denote Sn and Te atoms, respectively.

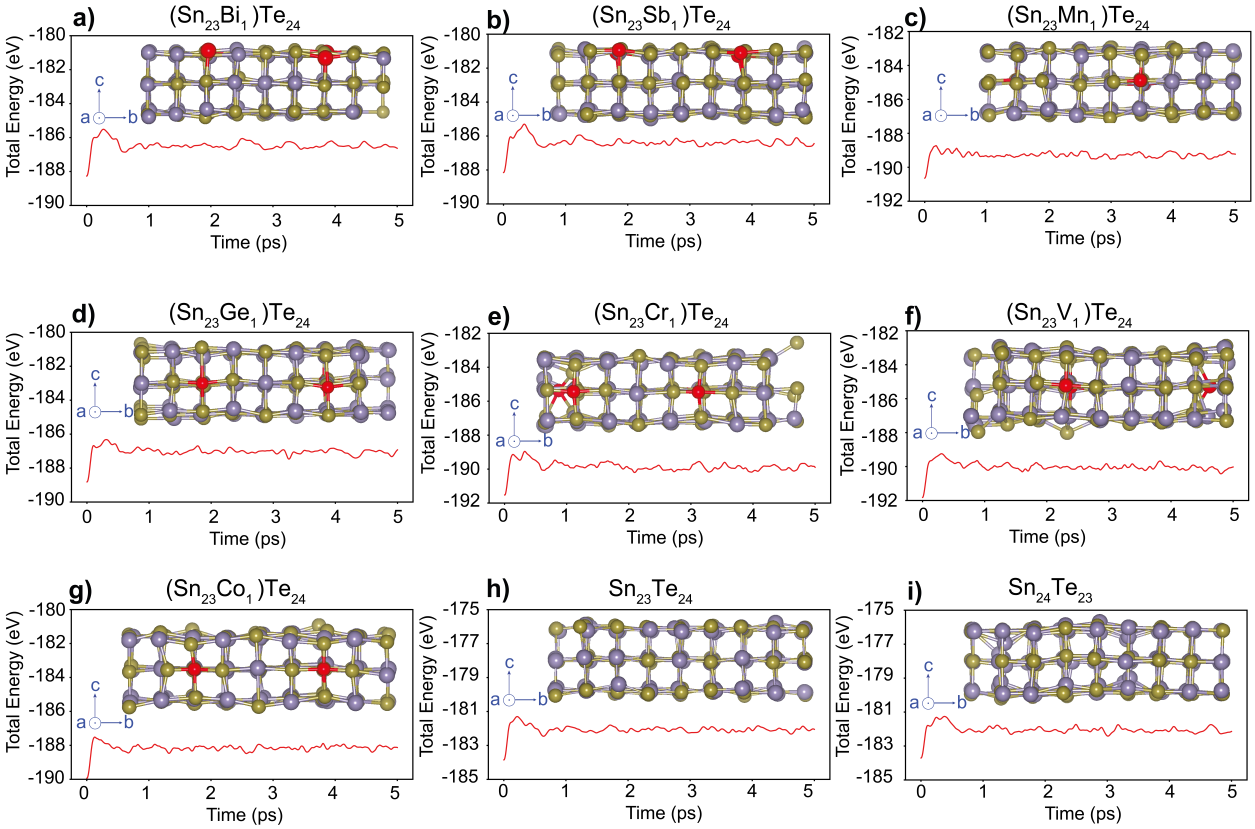

Figure S3. AIMD total-energy evolution (red) over 5 ps for cubic SnTe configurations: (a–g) doped, and (h,i) vacancy-defective. Insets show representative atomic snapshots; purple, gold, and red spheres denote Sn, Te, and dopant atoms, respectively.

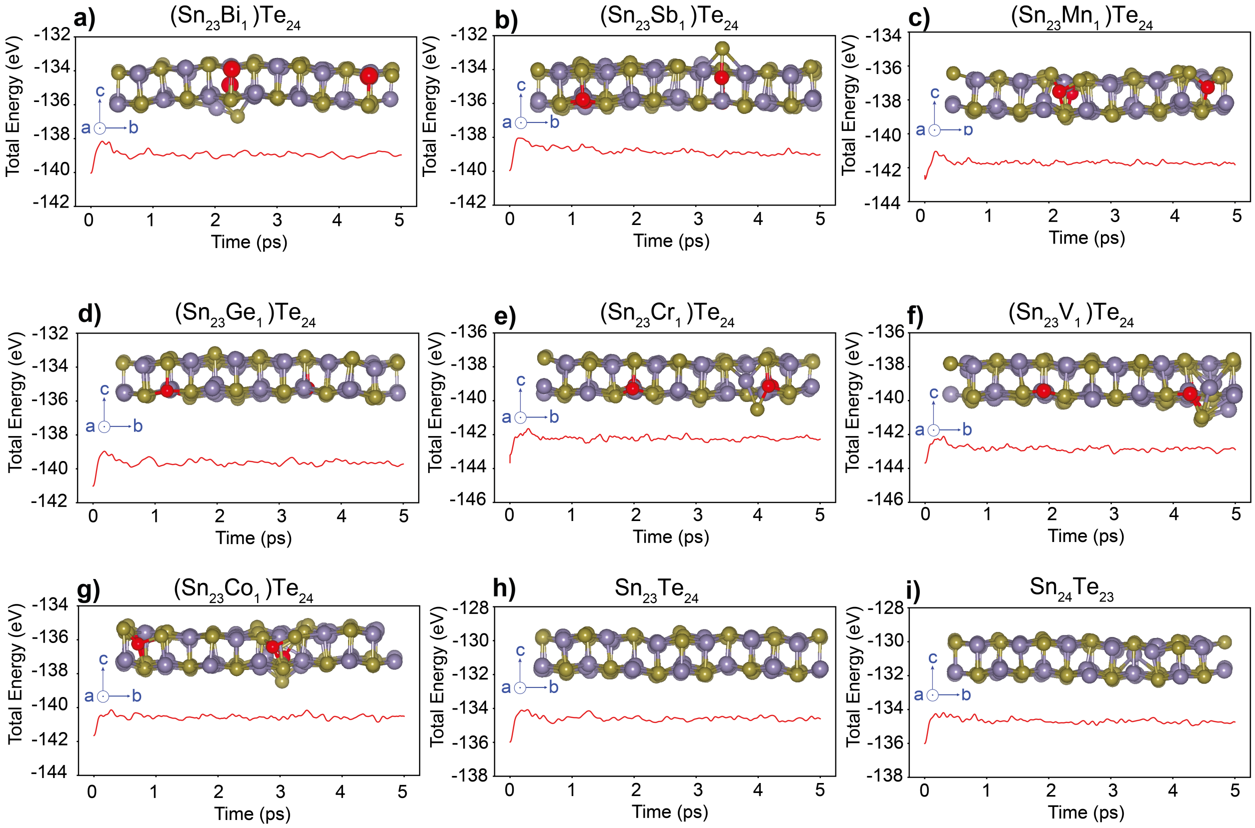

Figure S4. AIMD total-energy evolution (red) over 5 ps for γ-SnTe configurations: (a–g) doped, and (h,i) vacancy-defective. Insets show representative atomic snapshots; purple, gold, and red spheres denote Sn, Te, and dopant atoms, respectively.

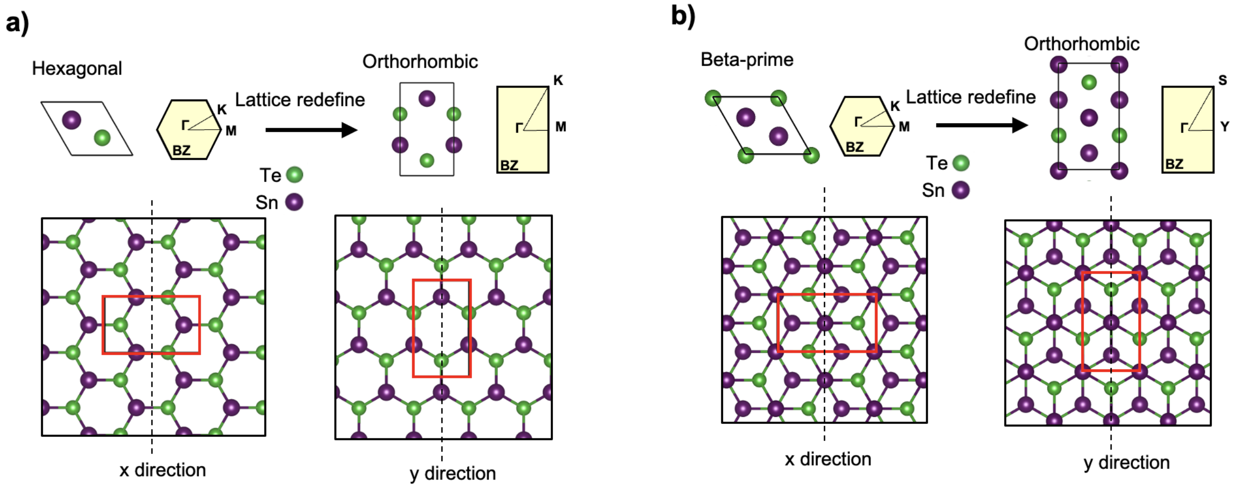

Figure S5. Lattice redefinition of (a) hexagonal and (b) β' SnTe into equivalent orthorhombic supercells. Sn (purple) and Te (green) atoms are shown, with zigzag and armchair directions indicated by dashed lines. Red rectangles mark the redefined orthorhombic unit cells.

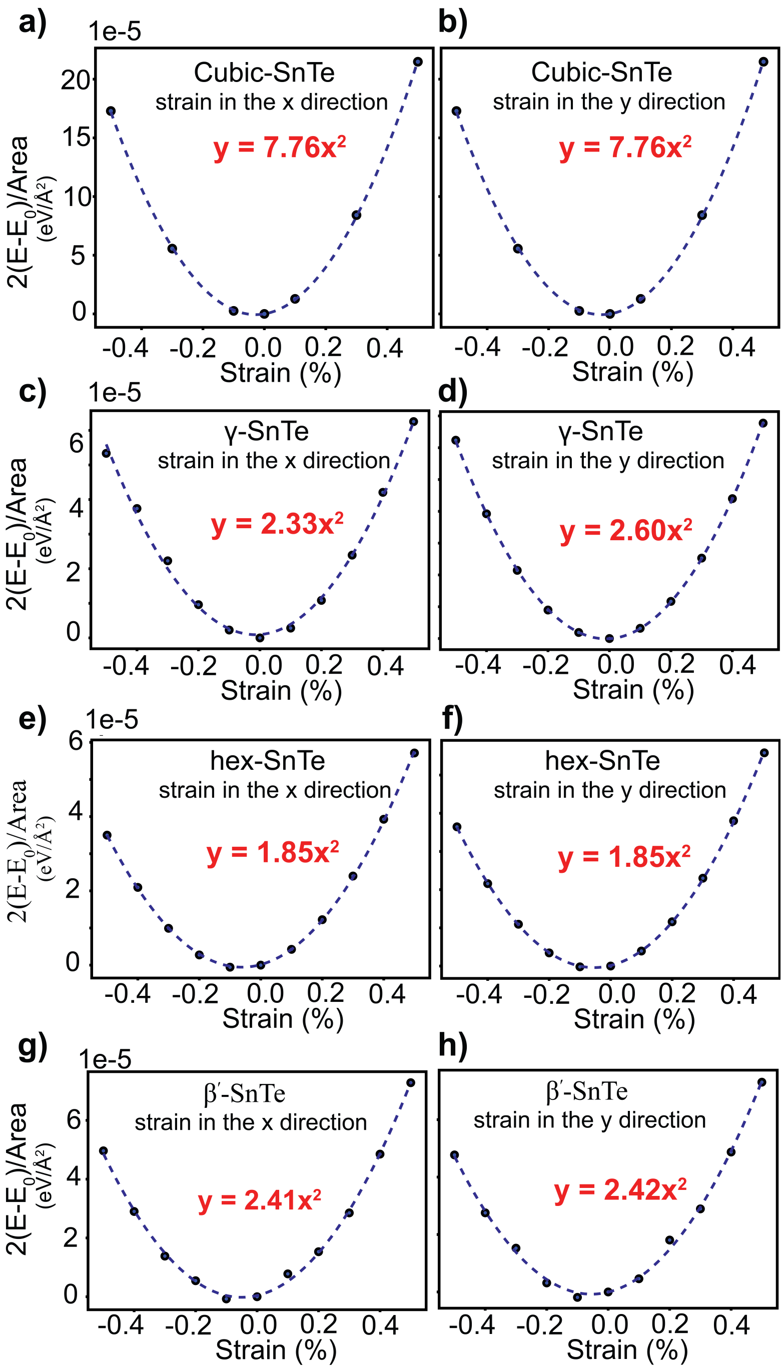

Figure S6. Calculated strain–energy relationships for (a,b) cubic-, (c,d) γ-, (e,f) hexagonal-, and (g,h) β'-SnTe monolayers under uniaxial strain along the x (left) and y (right) directions.

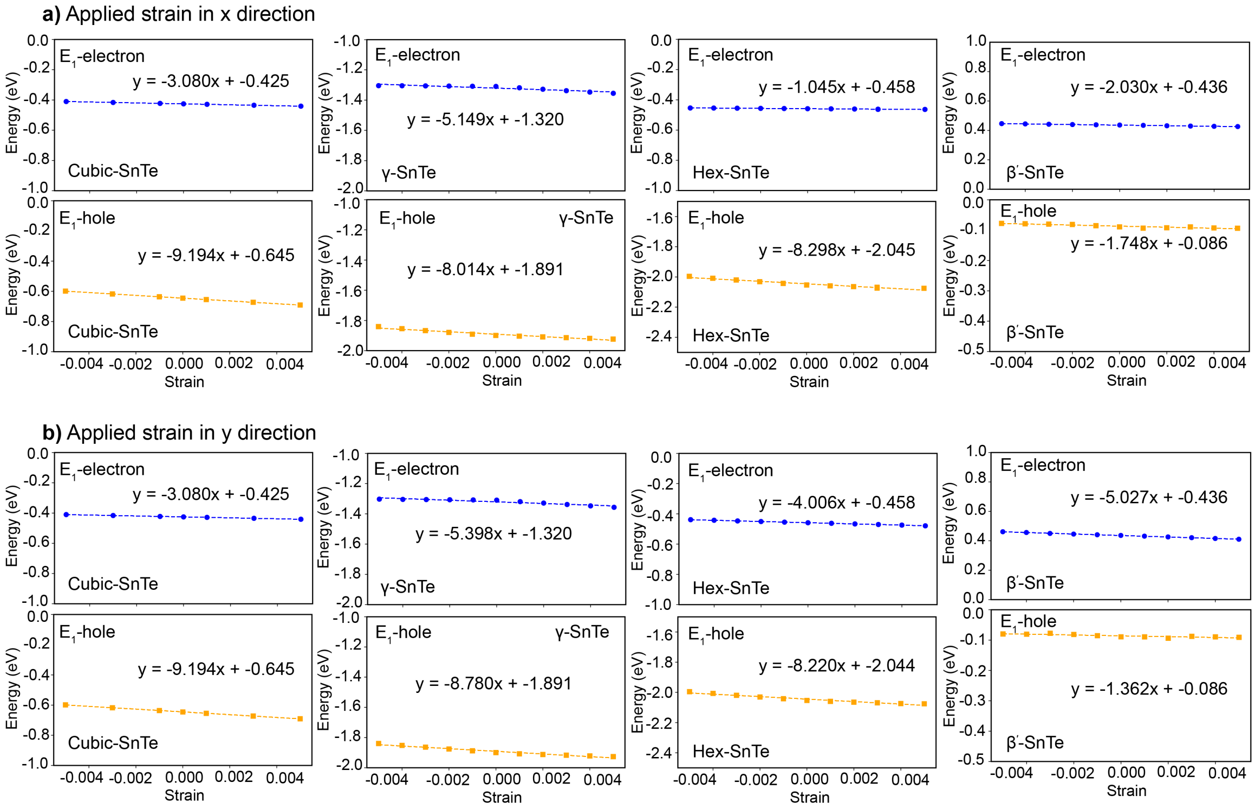

Figure S7. Variation of deformation potential constants (E_1) for electrons (top row) and holes (bottom row) in (a) x and (b) y directions of Cubic, γ-, hexagonal, and β'-SnTe. Linear fits (dashed lines) are shown along with the corresponding fitting equations, demonstrating the strain dependence of band edge energies used for carrier mobility calculations.

Table 1: Calculated total energies from spin-unpolarized (ISPIN = 1) and spin-polarized (ISPIN = 2) calculations for different dopants at inequivalent sites in cubic SnTe. The total magnetic moment of the defective supercell M is reported only for the spin-polarized calculations (ISPIN = 2), whereas ISPIN = 1 corresponds to a non-spin-polarized calculation. Bold entries correspond to the most energetically favorable (lowest E_{tot}) configuration for each dopant.

Dopant	Site	ISPIN = 1 E_{tot} (eV)	ISPIN = 2 E_{tot} (eV)	ISPIN = 2 M (μ_B)
Bi	**site 1**	-190.36434421	**-190.38523440**	**0.607**
	site 2	-190.36433815	-190.38520899	0.607
	site 3	-190.30816544	-190.30815421	NM
	site 4	-190.30817442	-190.30816535	NM
Sb	**site 1**	-190.25650503	**-190.28538372**	**0.489**
	site 2	-190.25650567	-190.28538352	0.489
	site 3	-190.16998494	-190.17000257	NM
	site 4	-190.16998493	-190.17000258	NM
Mn	site 1	-193.09336723	-195.31515252	4.450
	site 2	-193.09406187	-195.31507044	4.456
	**site 3**	-193.48090836	**-195.46526273**	**4.467**
	site 4	-193.48088300	-195.46525292	4.468
Ge	site 1	-190.89337261	-190.89345393	NM
	site 2	-190.89337260	-190.89345441	NM
	site 3	-190.97267171	-190.97277808	NM
	**site 4**	-190.97267345	**-190.97277832**	**NM**
Cr	site 1	-193.46147305	-195.42758965	3.661
	site 2	-193.45753658	-193.95127707	0.043
	site 3	-193.72958666	-195.57151216	3.669
	**site 4**	-193.72959773	**-195.57159219**	**-3.670**
V	site 1	-193.64610218	-194.71761530	2.682
	site 2	-193.64958794	-194.71761227	2.682
	**site 3**	-194.00200950	**-195.14586128**	**2.679**
	site 4	-194.06469388	-195.14586115	2.679
Co	site 1	-192.05766469	-192.47392611	2.522
	site 2	-192.27315510	-192.23725260	2.462
	site 3	-192.28190410	-192.50392889	2.555
	**site 4**	-192.28199503	**-192.50412541**	**2.554**

Table 2: Calculated total energies from spin-unpolarized (ISPIN = 1) and spin-polarized (ISPIN = 2) calculations for different dopants at inequivalent sites in γ-SnTe. The total magnetic moment of the defective supercell M is reported only for the spin-polarized calculations (ISPIN = 2), whereas ISPIN = 1 corresponds to a non-spin-polarized calculation. Bold entries correspond to the most energetically favorable (lowest E_{tot}) configuration for each dopant.

Dopant	Site	ISPIN = 1 E_{tot} (eV)	ISPIN = 2 E_{tot} (eV)	ISPIN = 2 M (μ_B)
Bi	**site 1**	-141.57901417	**-141.57925071**	**NM**
	site 2	-141.57857937	-141.57891833	NM
Sb	site 1	-141.49221598	-141.49246563	NM
	**site 2**	-141.49240741	**-141.49257609**	**NM**
Mn	**site 1**	-144.67409221	**-146.98964582**	**4.427**
	site 2	-144.67408934	-146.98961736	4.427
Ge	site 1	-142.51622370	-142.51592254	NM
	**site 2**	-142.51617199	**-142.51675773**	**NM**
Cr	site 1	-145.01943901	-146.99311740	3.62
	**site 2**	-145.01975933	**-146.99340062**	**3.62**
V	site 1	-145.38650693	-146.43023207	2.679
	**site 2**	-145.39188676	**-146.43044151**	**2.679**
Co	**site 1**	-143.40883989	**-143.97285206**	**2.523**
	site 2	-143.40920631	-143.97276679	2.523

Table 3: Calculated total energies from spin-unpolarized (ISPIN = 1) and spin-polarized (ISPIN = 2) calculations for Sn and Te vacancies at inequivalent sites in cubic SnTe. The total magnetic moment of the defective supercell M is reported only for the spin-polarized calculations (ISPIN = 2), whereas ISPIN = 1 corresponds to a non-spin-polarized calculation. Bold entries correspond to the most energetically favorable (lowest E_{tot}) configuration for each vacancy type.

Defect	Site	ISPIN = 1 E_{tot} (eV)	ISPIN = 2 E_{tot} (eV)	ISPIN = 2 M (μ_B)
V_{Sn}	**site 1**	**-185.93243102**	-185.93232534	**NM**
	site 2	-185.93232615	-185.93232394	NM
	site 3	-185.61277156	-185.61288428	NM
	site 4	-185.61286323	-185.61282024	NM
V_{Te}	site 1	-185.55662065	-185.55662611	NM
	site 2	-185.55662835	-185.55646183	NM
	site 3	-186.00595890	-186.00599153	NM
	**site 4**	-186.00595743	**-186.00599179**	**NM**

Table 4: Calculated total energies from spin-unpolarized (ISPIN = 1) and spin-polarized (ISPIN = 2) calculations for Sn and Te vacancies at inequivalent sites in γ-SnTe. The total magnetic moment of the defective supercell M is reported only for the spin-polarized calculations (ISPIN = 2), whereas ISPIN = 1 corresponds to a non-spin-polarized calculation. Bold entries correspond to the most energetically favorable (lowest E_{tot}) configuration for each vacancy type.

Defect	Site	ISPIN = 1 E_{tot} (eV)	ISPIN = 2 E_{tot} (eV)	ISPIN = 2 M (μ_B)
V_{Sn}	site 1	-137.35737428	-137.35724657	NM
	**site 2**	-137.35722842	**-137.35740611**	**NM**
V_{Te}	**site 1**	**-137.42989619**	-137.42989231	**NM**
	site 2	-137.42984885	-137.42989540	NM
